# Regulating Ligand‐to‐Metal Charge Transfer in UiO‐66(Zr)‐NH_2_ With Site‐Isolated Cu^+^ Centers for Efficient Photocatalytic H_2_O_2_ Generation

**DOI:** 10.1002/smsc.70333

**Published:** 2026-07-30

**Authors:** Aneek Kuila, Khaled Dassouki, Aditya Swarup Lal, Ambili Ramanthrikkovil Variyam, Nirmalendu Sekhar Mishra, Eddy Dumas, Nadav Amdursky, Nathalie Steunou, Yaron Paz

**Affiliations:** ^1^ Department of Chemical Engineering Technion ‐ Israel Institute of Technology Haifa Israel; ^2^ UMR CNRS 8180 Institut Lavoisier de Versailles, Université Paris‐Saclay Versailles France; ^3^ Environmental Nanotechnology Laboratory Department of Environmental Science and Engineering Indian Institute of Technology (ISM) Dhanbad Jharkhand India; ^4^ Schulich Faculty of Chemistry Technion‐Israel Institute of Technology Haifa Israel; ^5^ Green Energy & Applied Research (G.E.A.R) Lab Department of Environmental Science and Engineering SRM University‐AP Amaravati Andhra Pradesh India; ^6^ Chemistry, School of Mathematical and Physical Sciences University of Sheffield Sheffield UK

**Keywords:** metal‐organic frameworks (MOFs), photocatalysis, site‐isolated Cu^+^ centers, transient infrared (TRIR) spectroscopy, UiO‐66(Zr)‐NH_2_

## Abstract

Site‐isolated metal centers within metal‐organic frameworks (MOFs) offer a promising strategy to regulate charge–transfer dynamics and enhance photocatalysis. Here, we investigate the influence of atomically dispersed Cu ions on the photophysics and photocatalytic activity of the zirconium‐based MOF UiO‐66(Zr)‐NH_2_. Cu was incorporated via wet impregnation, producing Cu@UiO‐66(Zr)‐NH_2_ composites with preserved crystallinity. Structural and spectroscopic analyses confirm that Cu is stabilized predominantly as site‐isolated Cu^+^ species interacting with the amino‐functionalized linker. Photoluminescence studies reveal that Cu incorporation introduces an additional ligand‐to‐Cu charge–transfer pathway that competes with the intrinsic ligand‐to‐cluster transfer to the Zr_6_O_4_(OH)_4_ nodes, resulting in strong photoluminescence quenching. Time‐resolved photoluminescence and transient infrared spectroscopy demonstrate that Cu sites efficiently capture photogenerated electrons, suppressing energy dissipation and accelerating charge‐separation processes. These electronic effects significantly enhance photocatalytic oxygen reduction for H_2_O_2_ production under simulated solar irradiation. The H_2_O_2_ yield upon using 5 mg of MOF increased from ∼1.5 µmol  h^−1^ for pristine UiO‐66(Zr)‐NH_2_ to ∼8.2 µmol  h^−1^ for the 7 wt% Cu‐loaded material. Mechanistic investigations indicate Cu^+^ centers promote oxygen adsorption and activation, favoring the two‐electron oxygen reduction pathway. This work demonstrates that site‐isolated Cu ions can modulate ligand‐to‐metal charge–transfer processes in MOFs, offering a rational strategy to design efficient photocatalysts.

## Graphical abstract

Incorporation of highly dispersed Cu species into UiO‐66(Zr)‐NH_2_ introduces a competing ligand‐to‐Cu charge–transfer pathway that suppresses charge recombination and enhances photocatalytic H_2_O_2_ production. Combined PL, TRPL, TRIR, and variable‐temperature FTIR analyses reveal Cu‐mediated modulation of excited‐state dynamics and oxygen reduction pathways and provide mechanistic insights into charge–transfer engineering in MOF photocatalysts.

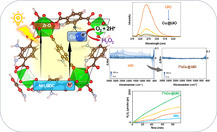

## 1 Introduction

Metal organic frameworks (MOFs) have become an important class of materials for photocatalysis because they offer an unusual combination of molecular‐level tunability and long‐range structural order. Their modular construction allows light‐harvesting linkers and catalytically active metal nodes to be integrated within a single crystalline framework, providing a platform to study and control charge generation, separation, and relaxation with high precision [1, 2]. Among the various MOF families, zirconium‐based frameworks such as UIO‐66 are particularly attractive due to their high chemical robustness, tolerance to defects, and stability under photochemical conditions [3, 4]. The amino‐functionalized derivative UiO‐66(Zr)‐NH_2_ is one of the most widely studied photoactive Zr‐MOFs. The introduction of the –NH_2_ group extends optical absorption into the visible region and gives rise to ligand‐centered excited states that can participate in photocatalytic reactions. Upon photoexcitation, these states may undergo ligand‐to‐cluster charge transfer (LCCT) to the {Zr_6_O_4_(OH)_4_} node [5]. While this process enables photochemical activity, it is often accompanied by rapid charge recombination and inefficient excited‐state utilization, which limits overall performance [6].

One strategy to overcome these limitations is the introduction of secondary metal centers that can selectively intercept photogenerated charge carriers. Redox‐active metals can act as electron or hole sinks, thereby altering the balance between radiative recombination, nonradiative decay, and charge separation [7]. Copper is particularly interesting in this context because of its accessible redox chemistry and strong affinity for nitrogen‐ and oxygen‐donor ligands [8]. The encapsulation of copper within MOFs has been previously reported; however, achieving precise control over the nature and oxidation state of the resulting Cu species remains challenging. Copper can exist in multiple forms, including Cu^2+^ cations, CuO_*x*_ clusters, or nanoparticles, often coexisting within the framework. This structural and chemical heterogeneity complicates their characterization and obscures the contribution of atomically dispersed species. Consequently, it becomes difficult to unambiguously interpret the photophysical behavior of Cu‐loaded MOFs. Stabilization of monovalent Cu^+^ within a rigid coordination environment offers an alternative route to harness copper's electronic properties without aggregation. Despite this promise, the impact of Cu incorporation on the excited‐state energy landscape, charge–transfer pathways, and vibrational relaxation of UiO‐66(Zr)‐NH_2_ has not been systematically resolved. In particular, it remains unclear how Cu affects the competition between ligand‐to‐Zr and ligand‐to‐metal charge transfer, and how this competition influences photoluminescence, excited‐state lifetimes, and photoinduced lattice dynamics.

In this work, we investigate the effect of site‐isolated Cu incorporation in UiO‐66(Zr)‐NH_2_ on its photophysical properties and photocatalytic behavior. By integrating structural characterization with photoluminescence (PL), time‐resolved infrared spectroscopy (TRIR), and variable‐temperature FTIR measurements, we elucidate how Cu^+^ species modify the excited‐state charge–transfer dynamics of the framework. Our results reveal that Cu incorporation introduces an additional ligand‐to‐Cu charge–transfer channel that competes with the intrinsic ligand‐to‐Zr charge transfer. This interaction generates electron‐rich Cu‐centers that act as oxygen adsorption and activation sites, promoting the formation of superoxide intermediates and selectively driving the two‐electron oxygen reduction pathway for H_2_O_2_ production.

These findings provide mechanistic insight into how site‐isolated metal ions can regulate charge–transfer pathways and catalytic activity in MOF‐based photocatalysts, offering a design strategy for improving photocatalytic oxygen reduction and solar‐driven H_2_O_2_ synthesis.

## 2 Results

### 2.1 Structural Characterization

**FIGURE 1:**
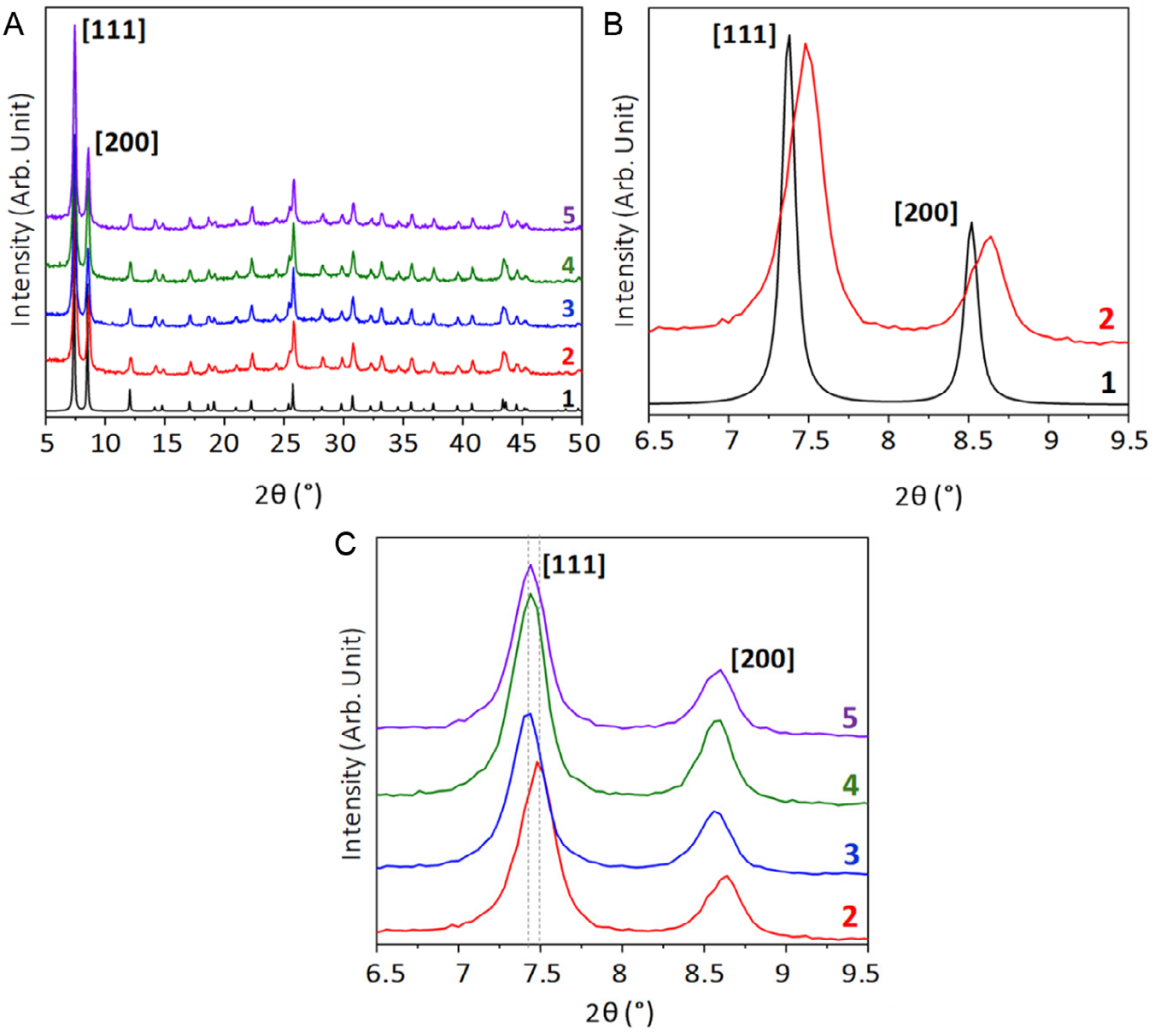
(A) Experimental PXRD patterns of UiO‐66(Zr)‐NH_2_ (2: red) and Cu@UiO‐66(Zr)‐NH_2_ composites with different Cu loadings 2 wt% (3: blue), 4 wt% (4: green), and 7 wt% Cu (5: purple) in comparison to the calculated pattern of UiO‐66(Zr)‐NH_2_ (1: black). (B) Zoom in section showing the calculated (1: black) and pristine UiO‐66(Zr)‐NH_2_ (2: Red). (C) Zoom in section showing pristine and the Cu‐loaded UiO‐66(Zr)‐NH_2_ composites.

Figure 1A shows the powder X‐ray diffraction (PXRD) patterns of pristine UiO‐66(Zr)‐NH_2_, the Cu‐loaded UiO‐66(Zr)‐NH_2_ composites with 2, 4, and 7 wt% Cu, together with the calculated pattern of UiO‐66(Zr)‐NH_2_. The PXRD pattern of UiO‐66(Zr)‐NH_2_ closely matches the calculated UiO‐66 structure, while exhibiting a slight shift of the diffraction peaks toward higher 2*θ* values (Figure 1B), which can be attributed to a minor lattice contraction. Upon Cu incorporation, all characteristic reflections of UiO‐66(Zr)‐NH_2_ are retained, indicating that the crystallinity and structural integrity of the MOF are preserved for all Cu loadings investigated. No additional diffraction peaks associated with crystalline copper or copper oxide phases are detected. A magnified view of the low‐angle region reveals a small but consistent shift of the [111] and [200] reflections toward lower 2*θ* values for all Cu‐containing samples compared to pristine UiO‐66(Zr)‐NH_2_ (Figure 1C), indicating a slight lattice expansion or local structural relaxation induced by the presence of Cu species within the framework.

**FIGURE 2:**
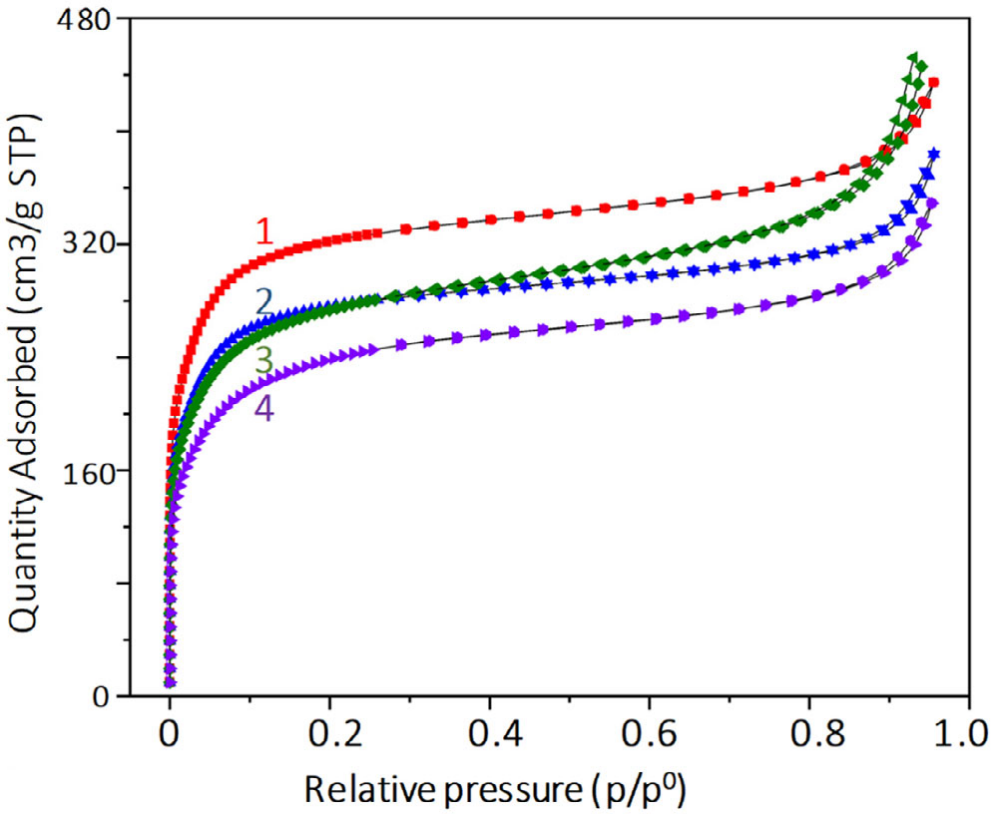
N_2_ adsorption–desorption isotherms of the MOFs. Pristine UiO‐66(Zr)‐NH_2_ (1: Red), 2 wt% (2: blue), 4 wt% (3: green), and 7 wt% Cu (4: purple).

Figure 2 shows the N_2_ adsorption–desorption isotherms of pristine UiO‐66(Zr)‐NH_2_ (a) and the Cu‐loaded UIO‐66(Zr)‐NH_2_ composites with 2 wt% (2), 4 wt% (3), and 7 wt% (4) Cu. All samples display Type‐I isotherms, characteristic of microporous materials, with a steep uptake at low relative pressures (*P*/*P*_0_ < 0.1), confirming the preservation of the intrinsic microporosity of the UiO‐66 framework after Cu incorporation. The small hysteresis loop at *p*/*p*° > 0.8 suggests the presence of additional textural porosity associated with interparticle voids, which is consistent with the nanosized and partially aggregated morphology of the composites. The BET surface area of pristine UiO‐66(Zr)‐NH_2_ is 1088 m^2^ g^−1^. Upon Cu incorporation, a gradual decrease in surface area is observed, with values of 940, 898, and 757 m^2^ g^−1^ for the 2, 4, and 7 wt% Cu‐loaded UiO‐66(Zr)‐NH_2_ samples, respectively. The micropore volume, determined by the t‐plot method, decreases from 0.35 cm^3^ g^−1^ for the pristine material to 0.29, 0.23, and 0.18 cm^3^ g^−1^ for the corresponding Cu‐containing samples. This progressive reduction in accessible micropore volume is consistent with partial pore filling or occupation of microporous cavities by Cu species while preserving the interconnected pore network of the UiO‐66 structure. Despite these changes, all Cu‐loaded materials retain significant residual porosity, indicating that the MOF framework remains largely accessible.

### 2.2 HRTEM Study

**FIGURE 3:**
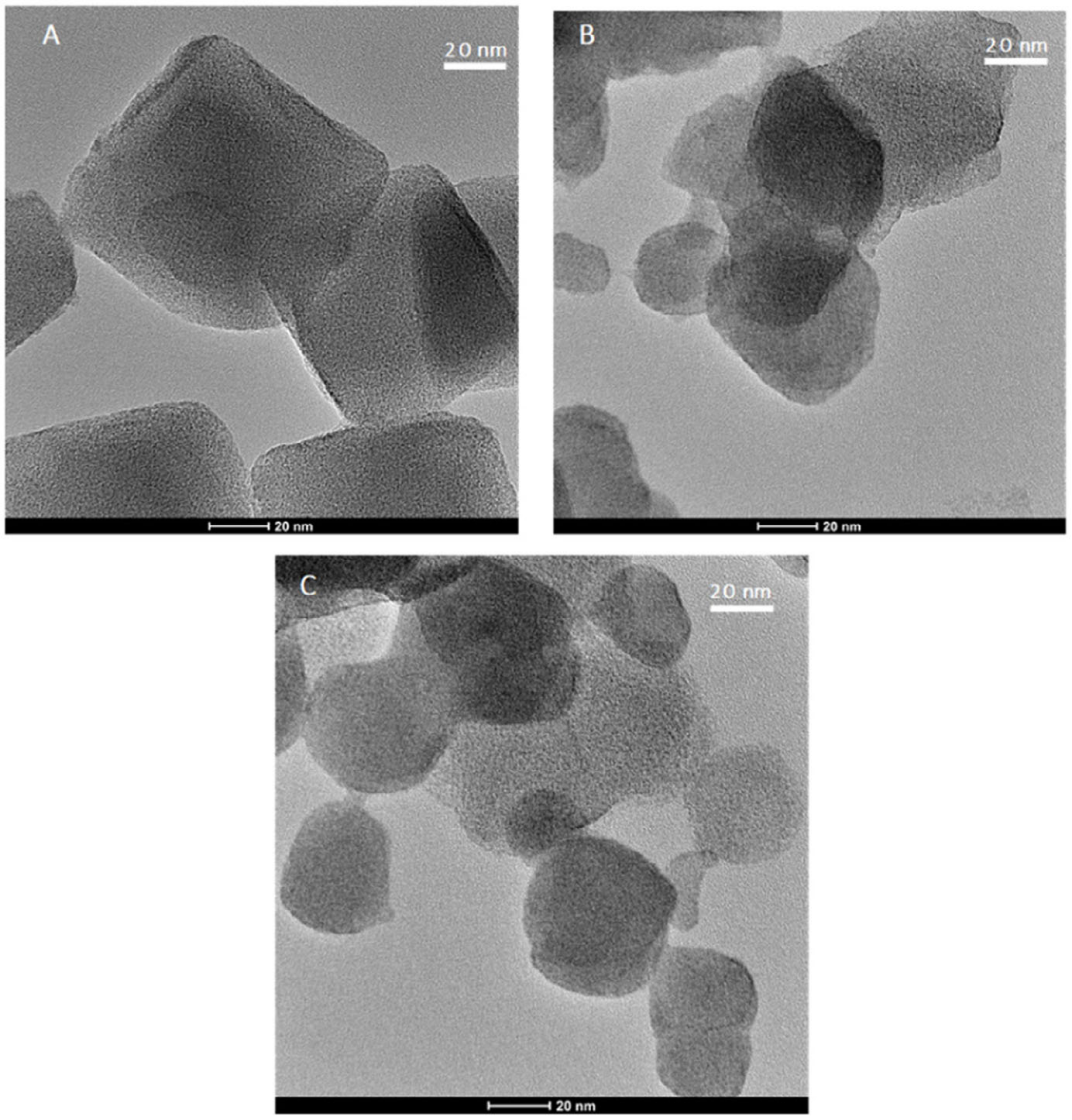
HRTEM Image of the (A) UiO‐66(Zr)‐NH_2_, (B) 2% Cu@UiO‐66(Zr)‐NH_2_, and (C) 7% Cu@UiO‐66(Zr)‐NH_2_.

The morphology of Cu‐modified UiO‐66(Zr)‐NH_2_ nanocrystals was investigated by combining transmission electron microscopy (TEM), high angle annular dark field in the scanning transmission electron microscopy mode HAADF–STEM and X‐ray energy dispersive spectrometer coupled with the STEM mode (i.e., STEM‐XEDS) in comparison to pure UiO‐66(Zr)‐NH_2_ (Figure 3A). 2% and 7% Cu@UiO‐66‐NH_2_ consist of spheroidal nanocrystals with an average size of about 30–50 nm (see Figures 3B,C, S1 and S2). Such particle morphology is fully consistent with that of pure UiO‐66(Zr)‐NH_2_. Elemental mapping images of pristine and Cu@UiO‐66(Zr)‐NH_2_ confirm the homogeneous distribution of Zr, O, C, N, and Cu throughout the entire MOF particles (Figures S1A,B and S2). No Cu‐rich aggregates or separate copper‐containing phases are observed within MOF nanocrystals (Figures 3 and S2). They are likely to be present as nanoscale clusters or site‐isolated Cu centers confined within the porous framework. Such Cu species are expected to be anchored in the UiO‐66(Zr)‐NH_2_ framework through coordination either with amino and/or OH groups of the {Zr_6_O_4_(OH)_4_} cluster.

### 2.3 Elemental Analysis

**FIGURE 4:**
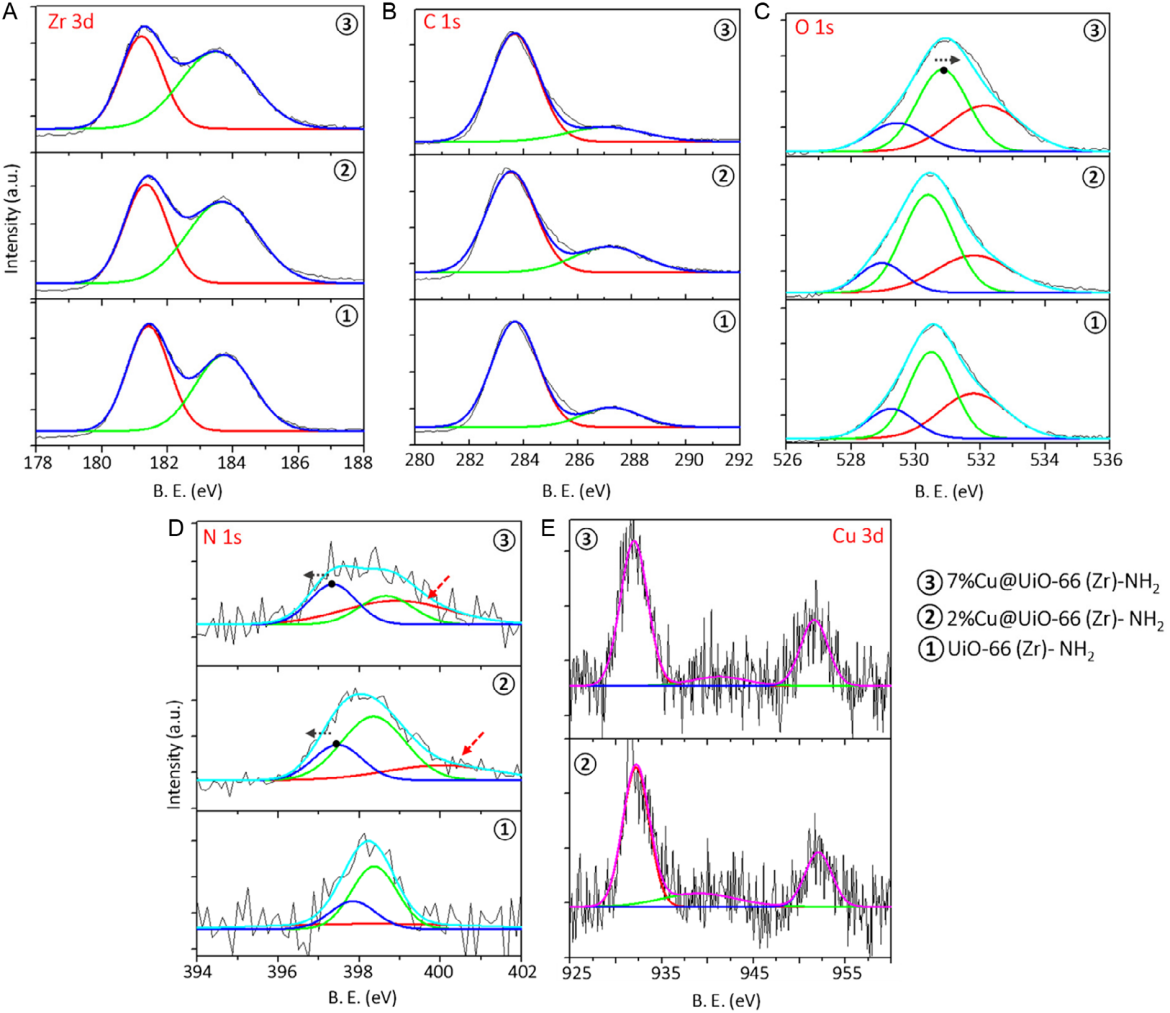
XPS spectra of UiO‐66(Zr)‐NH_2_ and 2 & 7 wt% UiO‐66(Zr)‐NH_2_.

The elemental composition and oxidation states of the UiO‐66 series were examined by X‐ray photoelectron spectroscopy (XPS) (Figure 4A–E). Pristine UiO‐66(Zr)‐NH_2_ exhibits two well‐resolved peaks at 181.3 and 183.9 eV, corresponding to Zr 3*d*_5/2_ and Zr 3*d*_3/2_, respectively (Figure 4A). These binding energies are characteristic of Zr^4+^ species in a fully oxidized coordination environment within the Zr–O clusters. Upon Cu modification, no measurable shifts in the Zr 3*d* binding energies are observed for 2%Cu@UiO‐66(Zr)‐NH_2_ and 7%Cu@UiO‐66(Zr)‐NH_2_, indicating that the electronic environment of the Zr centers remains largely unchanged. This implies that Cu incorporation does not disrupt the Zr–O framework and that the UIO‐66 backbone is preserved. The C 1s spectrum (Figure 4B) displays two dominant components at 283.8 eV, assigned to aromatic C—C/C=C, and 287.4 eV, corresponding to carboxylate O—C=O. These features remain unchanged upon Cu modification, confirming the chemical integrity of the organic linker backbone.

The O 1*s* spectrum (Figure 4C) was deconvoluted into three components. The peak at ∼529.1 eV is assigned to lattice oxygen (Zr—O—Zr). The component at ∼530.5 eV corresponds to metal–ligand bridging oxygen in M—O—C linkages (M=Zr). The higher‐binding‐energy peak at ∼532.0 eV is attributed to surface hydroxyl groups or physisorbed oxygen species. For pristine UIO‐66(Zr)‐NH_2_ and 2%Cu@UiO‐66(Zr)‐NH_2_, the metal–ligand component shows no measurable shift. For pristine UiO‐66(Zr)‐NH_2_ and 2%Cu@UiO‐66(Zr)‐NH_2_, the metal–ligand component shows no measurable shift. This is true also for the lattice oxygen (Zr—O—Zr) in the 7%Cu‐modified sample. The other two components in the oxygen peak of the highly copper‐doped UiO‐66 exhibit a binding‐energy upshift of ∼0.5 eV, indicating a reduction in electron density at the bridging oxygen atoms [9]. This shift reflects a modification of Zr—O—C covalency due to Cu‐induced electronic polarization, rather than structural rearrangement [10]. As no change in the Zr 3*d* binding energies is observed, this perturbation is attributed to Cu‐induced modification of the ligand–node interfacial environment, rather than the inorganic Zr—O cluster itself.

The N 1*s* region spans 396–402 eV (Figure 4D). Although the signal‐to‐noise ratio of the N 1*s* spectrum is relatively low, the peak deconvolution can still be performed with reasonable confidence. In pristine UiO‐66(Zr)‐NH_2_, the spectrum can be deconvoluted into two components. The peak at ∼397.5 eV is assigned to neutral amino nitrogen (—NH_2_) coordinated to the framework. The component at ∼398.2 eV corresponds to protonated or strongly hydrogen‐bonded nitrogen (—NH_3_^+^). In contrast, the N 1*s* spectra of the Cu‐modified UIO‐66(Zr)‐NH_2_ samples appear broadened, requiring three components for an adequate fit. In addition to the ‐NH_2_ and ‐NH_3_^+^ contributions, a new peak centered at ∼399.3–400.1 eV appears. This high‐binding‐energy component is attributed to nitrogen interacting with dopant Cu species (Cu–N) and/or a more oxidized nitrogen environment [11]. Its emergence provides direct evidence for Cu–ligand interaction through the amino functionality. Upon Cu incorporation, the –NH_2_ peak shifts to lower binding energy by ∼0.3 eV, independent of Cu loading, indicating increased electron density at the neutral amino nitrogen [12]. In contrast, the ‐NH_3_^+^ component shifts to higher binding energy by ∼0.6 eV at higher Cu loading, reflecting enhanced protonation or stronger hydrogen‐bonding interactions. Overall, the evolution of the N 1*s* spectra confirms that Cu is partially anchored through interactions with the amino groups of the NH_2_‐BDC linker, while the UiO‐66 framework remains intact.

In the 2% and 7% Cu‐modified samples, the Cu 2*p* region (Figure 4E) displays a well‐resolved doublet with Cu 2*p*_3_/_2_ at 932.1–932.2 eV and a spin–orbit splitting of ∼19.8 eV. A weak satellite feature (slightly above noise) is observed at ∼939 eV. This binding energy position, together with the absence of intense shake‐up satellites characteristic of Cu^2+^, is consistent with Cu predominantly existing in the +1‐oxidation state. No measurable shift in the Cu 2*p* binding energies is observed between the 2% and 7% Cu‐modified samples. This indicates that the chemical environment and binding nature of Cu remain unchanged over this loading range, suggesting that Cu is stabilized in a similar coordination environment within the UiO‐66 framework at both low and high Cu contents. It should be noted that, in view of the limitations inherent to conventional spectroscopic/diffraction methods in detecting such compounds, the presence of minute amounts of CuOx clusters, especially in samples having 7% Cu, cannot be ruled out.

### 2.4 Optical Absorption

**FIGURE 5:**
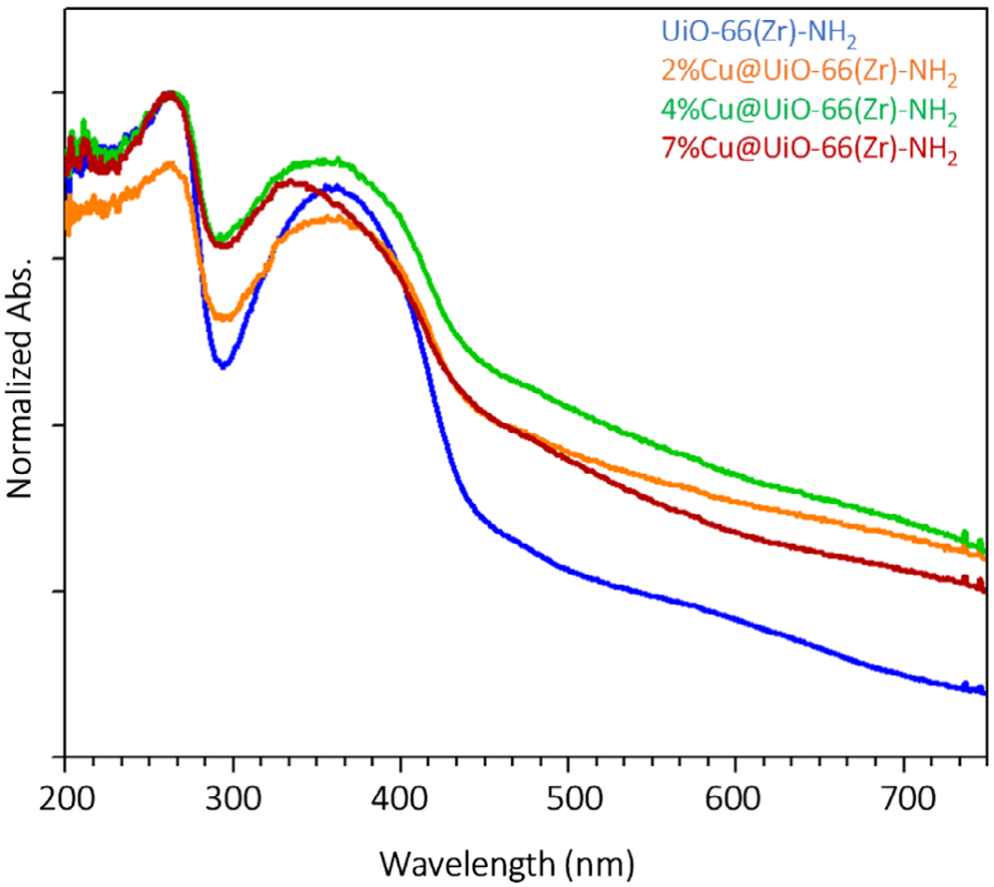
UV‐DRS spectra of UiO‐66(Zr)‐NH_2_ and Cu@UiO‐66(Zr)‐NH_2_ composites.

The solid‐state absorption of the MOFs is presented in Figure 5. Pristine UiO‐66(Zr)‐NH_2_ exhibits a well‐defined absorption edge in the ∼380–420 nm range, which originates from linker‐centered excitation of the amino‐functionalized terephthalate (NH_2_‐BDC) followed by ligand‐to‐cluster charge transfer (LCCT) to the {Zr_6_O_4_(OH)_4_} node. This behavior is consistent with previous reports on UiO‐66(Zr)‐NH_2_ [6, 13]. Upon Cu incorporation, the absorption peak at 360 nm is shifted to lower wavelengths (down to 340 nm in the case of 7% Cu). In contrast, the location of a second peak at 260 nm remains at the same wavelength. In parallel, a broad increase in visible‐light absorption between ∼420 and ∼750 nm without any specific peak is observed. This behavior indicates that Cu does not introduce a new, well‐defined optical transition, but rather generates a distribution of sub‐bandgap electronic states [14]. Such band‐edge tailing is characteristic of Urbach‐type behavior and reflects increased electronic disorder or the presence of shallow acceptor states [15].

The optical bandgaps of the MOFs were estimated from UV–Vis diffuse reflectance spectra using the Tauc method, assuming a direct band‐edge transition (Figure S3). The pristine UiO‐66(Zr)‐NH_2_ exhibits an apparent bandgap of approximately 2.83 eV, while the Cu‐modified samples show lower apparent values of ∼2.68, 2.59, and 2.61 eV for 2% Cu, 4% Cu, and 7% Cu loadings, respectively. This apparent reduction in bandgap should not be interpreted as a true intrinsic bandgap narrowing. Instead, it reflects the increasing contribution of sub‐bandgap absorption associated with Cu‐induced electronic states and band‐edge tailing [16, 17]. The non‐monotonic variation of the band‐gap values with Cu loading further supports this interpretation where dopant‐induced states obscure the true band edge.

### 2.5 Steady‐State Emission

**FIGURE 6:**
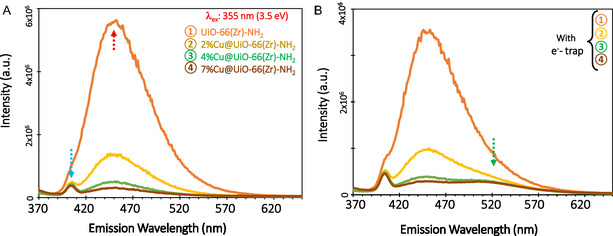
Steady‐state photoluminescence emission spectra of the various MOFs, recorded under 355 nm excitation: (A) in the absence of an electron scavenger and (B) in the presence of an electron scavenger. The arrows indicate the characteristic emission features centered at 405 (blue), 450 (red), and 510 nm (green).

The photoluminescence (PL) emission spectra recorded under 355 nm (3.5 eV) excitation are shown in Figure 6A. Pristine UiO‐66(Zr)‐NH_2_ exhibits a strong and broad emission band centered at  ∼450 nm (≈2.76 eV). This emission originates from ligand‐centered excited states of the amino‐functionalized terephthalate linker (NH_2_‐BDC). Partial ligand‐to‐cluster charge transfer (LCCT) to the {Zr_6_O_4_(OH)_4_} node also contributes to this emission [18]. Upon Cu incorporation, the PL intensity decreases systematically. The quenching follows the order 2% < 4% < 7% Cu‐modified UiO‐66(Zr)‐NH_2_. In contrast, the location of the emission maximum remains unchanged. The line shape remains almost the same, except for 7%Cu@UiO‐66(Zr)‐NH_2_ where a weak broadening is observed (Figure S4). This indicates that the emitting state remains ligand‐derived and that Cu does not introduce a new radiative center. The strong PL quenching by the copper may reflect the activation of efficient non‐radiative decay pathways. These pathways may arise from electron transfer to Cu sites, where Cu acts as an electron‐accepting or trapping center, suppressing radiative recombination [19].

A weak shoulder around ∼405 nm becomes more pronounced upon Cu loading (Figure 6A). Independent PL measurements of the free NH_2_‐BDC ligand, recorded under the same excitation wavelength (355 nm), show a distinct emission band at ∼407–410 nm (Figure S5), which can be attributed to a locally excited transition (*π*–π*) of NH_2_‐BDC [20]. The spectrum of the free NH_2_‐BDC ligand also reveals a broad lower‐energy emission band between 470 and 640 nm, peaked at 570 nm and containing a noticeable shoulder at 510 nm. These features are not observed in the spectrum of the MOF. The fact that the ∼405 nm peak appears both in the spectrum of the ligand and in the spectrum of the MOF suggests that this peak is ligand‐based. In the absence of copper, this relatively weak peak is overshadowed by the strong and broad 450 nm peak. The intensity's decrease of the 450 nm peak upon introducing copper disrupts the shadowing and makes this peak more pronounced. It should be noted that the intensity of the 405 nm peak is not attenuated by the presence of copper, suggesting that the copper is localized closer to the inorganic Zr—O subunits. This behavior is typical of systems in which electron‐accepting sites preferentially depopulate relaxed excited states rather than creating new radiative centers [21].

**FIGURE 7:**
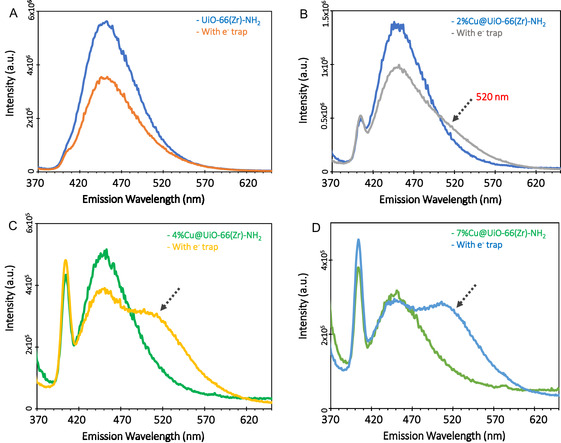
Comparative PL emission spectra of UiO‐66(Zr)‐NH_2_ and Cu@UiO‐66(Zr)‐NH_2_ composites under the influence of electron scavenger.

To further probe charge–transfer pathways, PL measurements were carried out in the presence of methyl viologen (MV^2+^), a well‐established electron scavenger (Figures 6B and 7A–D). For pristine UiO‐66(Zr)‐NH_2_, MV^2+^ addition primarily leads to a reduction in PL intensity without the appearance of new spectral features, as expected upon hindering radiative recombination (Figure 7A). In contrast, Cu@UiO‐66(Zr)‐NH_2_ samples display the emergence of a new broad emission band centered at ∼510 nm (≈2.43 eV) upon MV^2+^ addition (Figure 7B–D). Importantly, this emission coincides with the lower‐energy ligand emission band, already presented in free NH_2_‐BDC (Figure S5), demonstrating that this emission is ligand‐associated, rather than Cu or MV‐associated.

### 2.6 Time‐Resolved Photoluminescence

**FIGURE 8:**
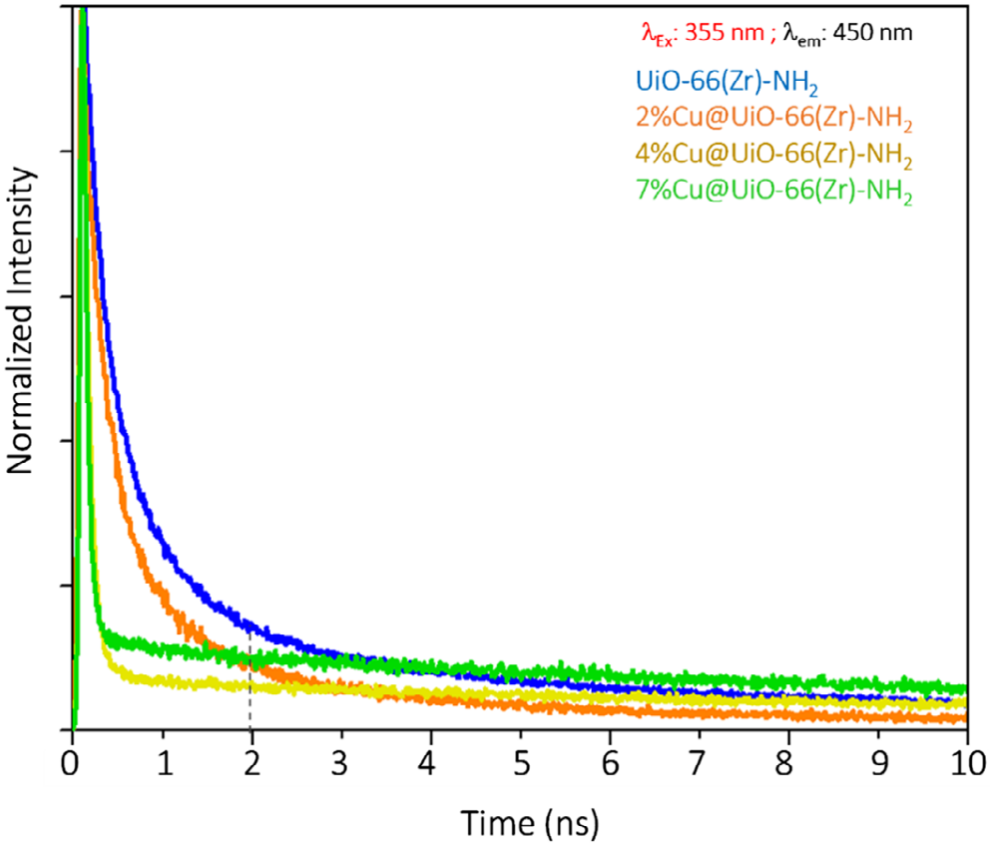
Time resolved PL emission of the MOFs.

To elucidate charge recombination dynamics and quantify excited‐state lifetimes, time‐resolved photoluminescence (TRPL) measurements were carried out for all samples (Figure 8). The samples were excited at 355 nm, and emission decays were monitored at 450 nm. All decay profiles are well described by a tri‐exponential model, characterized by three lifetimes (*τ*_1_, *τ*_2_, *τ*_3_, all in ns) with their corresponding amplitudes (*A*_1_, *A*_2_, *A*_3_). The extracted parameters are summarized in Table 1.

**TABLE 1:** Exponential fitting parameters of the various MOFs during TRPL measurements.

Sample	*A*_1_	*τ*_1_, ns	*A*_2_	*τ*_2_, ns	*A*_3_	*τ*_3_, ns
UiO‐66(Zr)‐NH_2_	0.90	0.15	0.42	0.61	0.12	4.39
2%Cu@UiO‐66(Zr)‐NH_2_	0.92	0.07	0.28	0.68	0.17	3.86
4%Cu@UiO‐66(Zr)‐NH_2_	5.68	0.04	0.12	0.16	0.07	9.29
7%Cu@UiO‐66(Zr)‐NH_2_	9.15	0.03	0.03	0.45	0.11	10.30

The decay component *τ*_1_ corresponds to fast nonradiative relaxation processes associated by deexcitation from the {Zr_6_O_4_(OH)_4_} inorganic building unit (IBU) to the linker, whereas *τ*_3_ represents a slow decay component. As portrayed in Table 1, the addition of copper increased significantly increased the nonradiative processes as indicated by the faster initial decay (first component) and its increased relative amplitude of the first component while slightly decreasing *τ*_1_. In parallel, the presence of copper did not alter the amplitude of the third component; however, it increased its time constant *τ*_3_, which means that the long‐lived component lives for longer timescales upon the addition of copper. The effect of copper on the fast component, together with the decrease in PL intensity (Figure 6), suggests the opening of a nonradiative channel that collects excited electrons quickly and efficiently on copper sites. The effect on the long‐lived slow component may suggest the presence of a second mechanism that gradually spills electrons back to the IBU, from where they may de‐excite, releasing 450 nm photons.

### 2.7 Steady‐State FTIR

**FIGURE 9:**
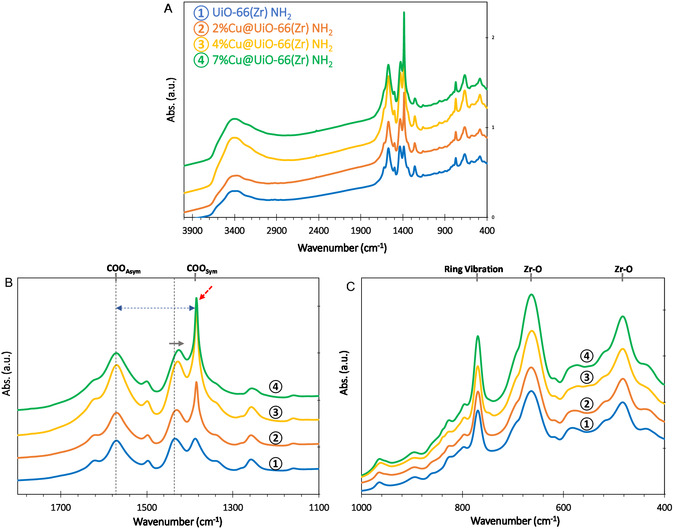
Steady State FTIR spectra of the various UiO‐66(Zr)‐NH_2_ MOFs. (A) whole spectra (B) zoom in at 1100–1800 cm^−1^ region, and (C) zoom in at 400–1000 cm^−1^ region. (1) UiO‐66(Zr)‐NH_2_, (2) 2%Cu@UiO‐66(Zr)‐NH_2_, (3) 4% Cu@UiO‐66(Zr)‐NH_2_, and (4) 7% Cu@UiO‐66(Zr)‐NH_2_.

The steady‐state FTIR spectra of the MOF samples are shown in Figure 9A. Pristine and Cu‐modified UiO‐66(Zr)‐NH_2_ display nearly identical spectral features, indicating that the framework structure is retained after Cu incorporation. The bands at ∼440 and ∼630 cm^−1^ are assigned to Zr–O stretching vibrations, while the band at ∼780 cm^−1^ originates from vibrations of the aromatic linker. In the carboxylate region, all samples exhibit a symmetric carboxylate stretching doublet, *ν*_s_(COO^−^), at ∼1380 and ∼1430 cm^−1^, together with the asymmetric stretching vibration *ν*_as_ (COO^−^) at ∼1580 cm^−1^. The difference between these modes (Δ*ν *= *ν*_as_‐*ν*_s_) provides information on the coordination environment of the carboxylate groups [22]. Evidently. the component at ∼1380 cm^−1^ corresponds to monodentate coordination, while the band at ∼1430 cm^−1^ is associated with bidentate coordination within the framework. The N—H bending vibration appears at ∼1260 cm^−1^, and the bands at ∼3367 and ∼3453 cm^−1^ correspond to the symmetric and asymmetric stretching modes of the NH_2_ groups.

Figure 9B,C show enlarged views of selected regions of the spectrum in Figure 9A. After Cu incorporation, no additional bands appear in the 400–1000 cm^−1^ region (Figure 9C), and no band splitting is detected. The preservation of these low‐frequency features indicates that the Zr–carboxylate coordination environment remains unchanged. Changes are observed only in the 1100–2000 cm^−1^ region (Figure 9B). In the Cu‐modified samples, the *ν*_s_(COO^−^) region becomes more pronounced. The intensity of the ∼1380 cm^−1^ band increases progressively with increasing Cu content, while the ∼1430 cm^−1^ component shows a slight shift toward lower wavenumber with nearly constant intensity. In contrast, the *ν*_as_ (COO^−^) band at ∼1578 cm^−1^ remains essentially unchanged. The enhanced intensity of the ∼1380 cm^−1^ band indicates a greater contribution from carboxylate groups exhibiting monodentate character, whereas the bidentate component remains largely unaffected. Alternatively, the increased intensity may arise from enhanced infrared oscillator strength due to higher polarizability of the carboxylate group in the presence of Cu species [9]. The small shift of the *ν*_s_ (COO^−^) vibration toward lower wavenumber suggests a reduction in the effective C—O force constant caused by Cu‐induced electronic polarization. Despite these spectral changes, the absence of band splitting and the stability of the *ν*_as_ (COO^−^) mode indicate that the overall coordination geometry of the carboxylate groups remains unchanged. These observations confirm that the structural integrity of the UiO‐66(Zr)‐NH_2_ framework is maintained after Cu incorporation. The spectral variations are therefore attributed to electronic perturbations within the linker environment rather than modifications of the Zr–carboxylate coordination structure, consistent with the XPS results indicating electrostatic interaction between Cu^+^ species and the organic linker [23].

### 2.8 Transient IR Analysis

**FIGURE 10:**
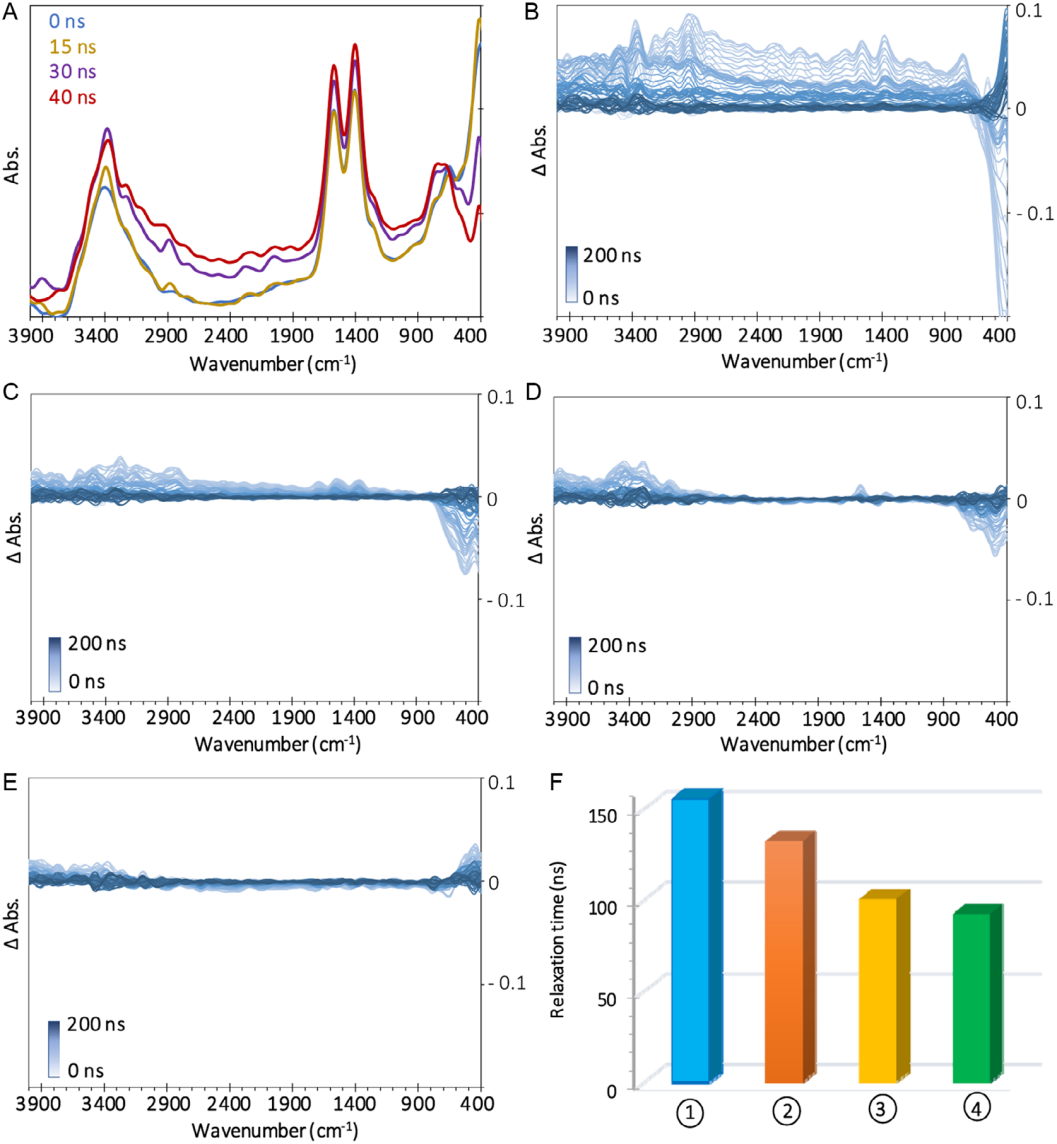
(A) Transient IR absorbance spectra of UiO‐66(Zr)‐NH_2_. TRIR ΔAbs spectra of (B) UiO‐66(Zr)‐NH_2_, (C) 2%Cu@UiO‐66(Zr)‐NH_2_, (D) 4%Cu@UiO‐66(Zr)‐NH_2_, (E) 7%Cu@UiO‐66(Zr)‐NH_2_ under 355 nm excitation, and (F) Post excitation relaxation time of the MOFs (1, UiO‐66(Zr)‐NH_2_ (blue); 2, 2%Cu@UiO‐66(Zr)‐NH_2_ (orange); 3, 4%Cu@UiO‐66(Zr)‐NH_2_ (yellow); 4 7%Cu@UiO‐66(Zr)‐NH_2_ (green)).

Figure 10A presents the time evolution of the transient infrared spectra of the MOFs following photoexcitation. Figure 9B–E shows the corresponding transient difference spectra (ΔAbs), calculated as ΔAbs = Abs_t_ – Abs_0_, where Abs_0_ is the pre‐excitation spectrum and Abs_t_ is the spectrum recorded at defined delay times. The spectra are shown for pristine UiO‐66(Zr)‐NH_2_ (Figure 9B) and for the 2%, 4%, and 7%Cu@UiO‐66(Zr)‐NH_2_ (Figure 10C–E), respectively. In pristine UiO‐66(Zr)‐NH_2_, the earliest detectable transient response consists of a decrease in absorption intensity at ∼480 and ∼660 cm^−1^, assigned to Zr—O vibrational modes of the inorganic building unit. This decrease is accompanied by a broad, featureless increase in the spectral baseline extending over approximately 1100–4000 cm^−1^. The baseline increase reaches a maximum at ∼40 ns after excitation and subsequently decays. This decay correlates with the recovery and eventual positive ΔAbs contribution of the Zr—O bands.

The observed featureless broadband response (aka “IR‐blackening”) [24] indicates the presence of a miniband containing high density of states, enabling IR absorption across the whole spectral region. These states can be electronic in nature or representing localized pre‐dissociative vibrational energy states [25, 26]. Upon Cu incorporation, a pronounced modification of the transient response is observed. The intensity of the broadband “IR blackening” feature progressively decreases with increasing Cu content (Figures 10C–E and S6A–C). In parallel, the amplitude of all other transient spectral features is also attenuated as the Cu loading increases. This indicates that Cu incorporation suppresses the formation and/or population of the transient states responsible for the broadband absorption.

The temporal evolution of the transient IR signal further reflects the influence of Cu. In all samples, the onset of transient vibrational changes occurs at ∼12–14 ns after excitation. However, the persistence of the transient response shortens systematically with increasing Cu content. In pristine UiO‐66(Zr)‐NH_2_, the transient signal remains detectable up to ∼155–160 ns. In contrast, the decay becomes progressively faster in Cu‐modified samples, with signal persistence reduced to ∼132 ns (2% Cu), ∼100 ns (4% Cu), and ∼92 ns (7% Cu) (Figure 10F). This monotonic decrease in transient lifetime demonstrates that increasing Cu loading enhances relaxation kinetics and promotes more efficient deactivation pathways.

It should be noted that excitation was performed using a pulsed laser operating at 10 Hz. Under these conditions, the spectrum recorded at *t* = 0 ns corresponds to the residual signal collected 100 ms after the preceding excitation pulse. In the present measurements, this residual signal was negligible. This was verified by comparing the *t* = 0 spectrum under excitation conditions with the spectrum acquired while blocking the excitation beam, which showed no detectable difference.

The transient change in the whole baseline suggests that the response reflects electronic or vibronic relaxation processes coupled to the lattice. It closely resembles the IR‐blackening effect observed for pristine NH_2_‐BDC ligand (Figure S7A,B) [27], where a featureless increase in mid‐IR absorption is attributed to electronic transitions between densely packed localized states. The baseline elevation partially masks transient vibrational signatures associated with individual functional groups. It should be noted that BDC alone (i.e., without the NH_2_ group) does not show this phenomenon (Figure S7D) so that the excited state leading to the IR blackening effect seems to be due to the NH_2_ group of the ligand.

To further probe the role of charge carriers, ΔAbs spectra were recorded in the presence of the electron scavenger methyl viologen (Figure S8A–D). Introduction of the scavenger led to complete suppression of transient absorption features in the 500–2800 cm^−1^ range, demonstrating that photoexcited electrons are the primary contributors to the observed TRIR response. Notably, new, very strong undulations in the transient spectra between ∼2950 and ∼3400 cm^−1^ emerged in the presence of the scavenger. These undulations are typical for MV and disappear when AgNO_3_ is used as an electron scavenger. These features are most pronounced for the 2% Cu‐modified sample and are progressively weaken at higher Cu loadings (4% and 7% Cu), reflecting differences in charge‐separation efficiency and electron‐trapping behavior.

### 2.9 Elevated Temperature FTIR

**FIGURE 11:**
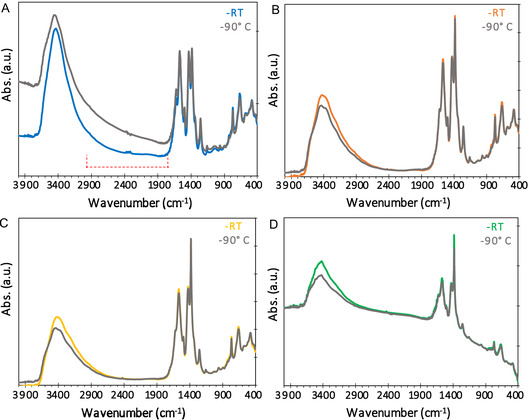
FTIR spectra of (A) UiO‐66(Zr)‐NH_2_, (B) 2%Cu@UiO‐66(Zr)‐NH_2_, (C) 4%Cu@UiO‐66(Zr)‐NH_2_, and (D) 7%Cu@UiO‐66(Zr)‐NH_2_ measured from room temperature (RT) to 90°C.

To distinguish between specific transient spectral changes in the excited state and thermally induced IR spectral changes in the ground state, in situ variable‐temperature FTIR measurements were performed on pristine and on Cu‐modified UiO‐66(Zr)‐NH_2_ (Figure 11A–D). For all materials, heating up to 90°C did not cause significant changes in the peak positions or line shapes of the characteristic vibrational bands, confirming that the UiO‐66(Zr)‐NH_2_ framework remains structurally stable over this temperature range.

The broad band at 2900–3700 cm^−1^ is characteristic for hydrogen bonding between OH groups and is likely to originated from adsorbed water. At room temperature, the intensity of this band decreases with increasing Cu content, indicating a lower amount of adsorbed water on the MOF surface. This trend is followed by the change in the specific surface area of the various MOFs (see above, structural characterization). As expected, increasing the temperature reduced the intensity of this peak in all types of materials, following some water desorption.

**FIGURE 12:**
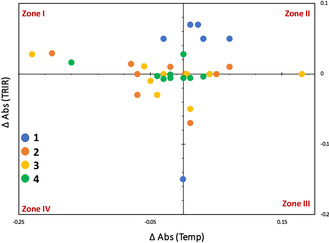
IR ΔAbs in TRIR measurements versus IR ΔAbs in in situ heating measurements of UiO‐66(Zr)‐NH_2_ MOFs: 1, UiO‐66(Zr)‐NH_2_ (blue); 2, 2%Cu@UiO‐66(Zr)‐NH_2_ (orange); 3, 4%Cu@UiO‐66(Zr)‐NH_2_ (yellow); and 4, 7%Cu@UiO‐66(Zr)‐NH_2_ (green).

The mechanistic origin of the distinct thermal responses was clarified by comparing the thermally induced spectral changes with the TRIR data (Figure 12). Here, the thermal ΔAbs is plotted against the TRIR ΔAbs, measured 40–45 ns after excitation. In such correlation plots, data points located in zone II and IV may indicate correlation between thermal effects and TRIR signals, ideally aligned along a straight line. This behavior reflects spectral changes arising from ground‐state heating. In contrast, data points located in zone I and III represent a situation in which the thermal changes and the TRIR changes are in opposite direction.

For pristine UiO‐66(Zr)‐NH_2_, several data points are scattered in Zone‐ II, indicating that a fraction of the observed TRIR response originates from thermal heating effects in the ground state. In contrast, for Cu‐modified UiO‐66(Zr)‐NH_2_, nearly all data points are distributed in Zone I and III. This distribution clearly demonstrates that the observed TRIR signals do not arise from thermal heating, but instead reflect vibrational changes associated with photoinduced electronic processes. The absence of correlation with thermal ΔAbs confirms that Cu incorporation effectively suppresses thermal contributions to the TRIR response. Importantly, this conclusion‐drawn from TRIR signals measured at ∼40 ns after excitation‐is fully consistent with the TRPL results, which show two distinct deexcitation mechanisms: a fast mechanism, in which the copper modified MOF is the fastest and a slow mechanism, in which the copper‐loaded MOFs are the slowest.

### 2.10 Photocatalytic Activity

**FIGURE 13:**
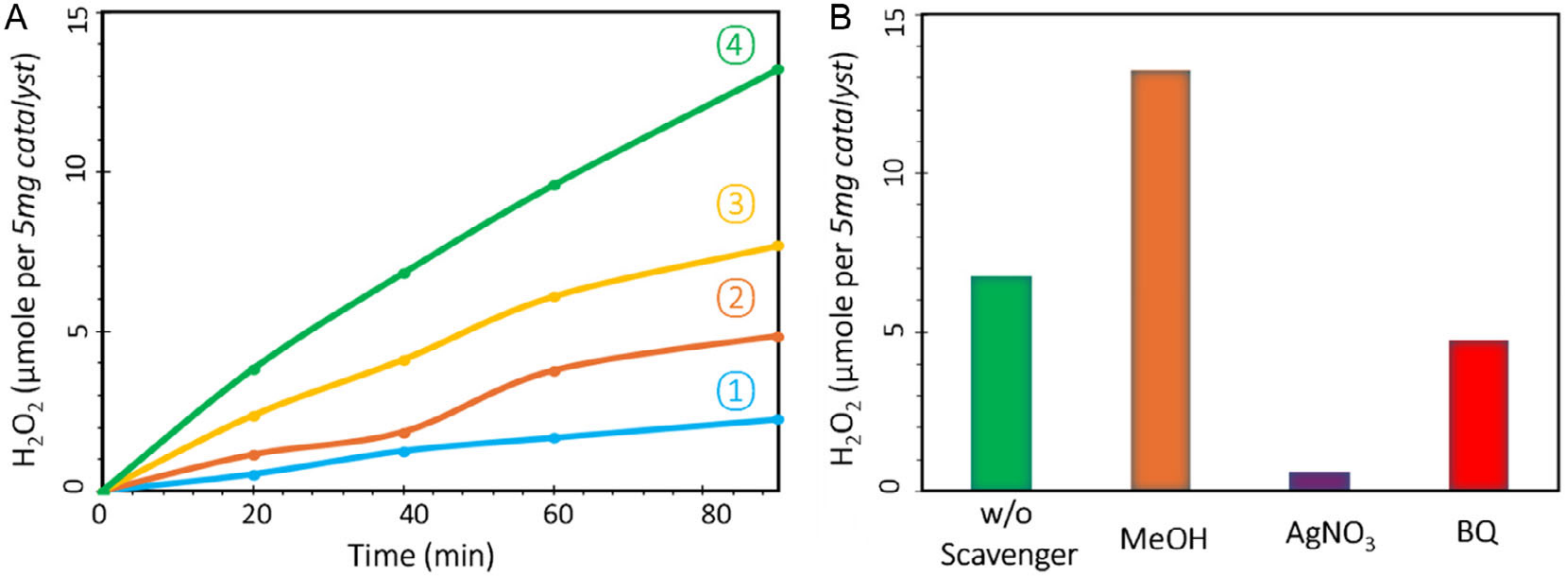
(A) Photocatalytic H_2_O_2_ production using UiO‐66(Zr)‐NH_2,_ (1: blue), 2%Cu@UiO‐66(Zr)‐NH_2_ (2: orange), 4%Cu@UiO‐66(Zr)‐NH_2_ (3: yellow), and 7%Cu@UiO‐66(Zr)‐NH_2_ (4: green) under simulated solar irradiation. (B) Influence of different radical scavengers on the photocatalytic H_2_O_2_ generation over 7%Cu@UiO‐66(Zr)‐NH_2_ after 90 min irradiation.

The photocatalytic H_2_O_2_ production of the MOF catalysts was evaluated in aqueous solution containing 10 vol% methanol as a hole scavenger under simulated solar irradiation for 90 min (Figure 13A). The reactions were conducted under an air atmosphere, as experiments performed under N_2_ atmosphere showed strongly suppressed H_2_O_2_ formation, confirming that dissolved O_2_ acts as the terminal electron acceptor. Under air, a clear and progressive enhancement in H_2_O_2_ generation was observed with increasing Cu incorporation into the UiO‐66(Zr)‐NH_2_ framework. The pristine UiO‐66(Zr)‐NH_2_ catalyst produced approximately 450 µmol g^−1^ of H_2_O_2_ after 90 min of irradiation. Upon Cu incorporation, the production yield increased significantly to approximately 970, 1540, and 2446 µmol g^−1^ for 2%Cu@UiO‐66(Zr)‐NH_2_, 4%Cu@UiO‐66(Zr)‐NH_2_, and 7%Cu@UiO‐66(Zr)‐NH_2_, respectively. Importantly, this improvement occurs despite a decrease in BET surface area upon Cu incorporation, indicating that the enhanced catalytic performance is not driven by increased surface accessibility. Instead, the pronounced increase in H_2_O_2_ productivity originates from electronic modulation induced by Cu incorporation, which promotes more efficient charge separation, suppresses electron–hole recombination, and facilitates selective electron transfer toward the oxygen reduction reaction (ORR). The increased availability of long‐lived photogenerated electrons consequently accelerates the two‐electron reduction pathway of O_2_ to H_2_O_2_. Nevertheless, excessive Cu incorporation might cause agglomeration, pore blockage, light shielding, or creation of recombination sites, which might have a negative impact on the photocatalytic activity. Therefore, the activity is not necessarily expected to increase indefinitely with increasing Cu loading [28].

To further elucidate the mechanism of H_2_O_2_ formation, a series of scavenger experiments were performed using the most active catalyst, 7%Cu@UiO‐66(Zr)‐NH_2_ (Figure 13B). The photocatalytic reaction was conducted under four different conditions: without scavengers, with methanol (10%) as a hole scavenger, with AgNO_3_ as an electron scavenger, and with benzoquinone (BQ) as a superoxide scavenger. In the absence of scavengers, the H_2_O_2_ yield after 90 min was approximately 6.7 µmol, when using 5 mg of MOF. This yield increased considerably to ∼13 µmol in the presence of methanol, indicating that water oxidation is the rate‐limiting half‐reaction under scavenger‐free conditions. The calculated quantum efficiency, defined hereby as the number of H_2_O_2_ moles *2 (each H_2_O_2_ molecule requires two electrons, hence two photons), multiplied by 100 and divided by number of impinging photons, gives 0.37% ± 0.05%, when using 5 mg of catalyst. This number is likely to be considerably higher if using larger amount of catalyst. Here, since a solar simulator was used (with a 420 nm filter), the number of impinging photons was calculated based on assuming an average photon energy of 2.75 eV. Comparing with other works is not trivial, since the experimental conditions vary from one study to the other, not to mention that many relevant publications fail to provide sufficient experimental details that may allow a true comparison. Having said that, one may compare with a work using a composite of UiO‐66‐NH_2_ and ZnIn_2_S_4_ reporting on a quantum efficiency of 1.79% when using four times the amonunt of catalyst that was used in our measurements [29]. Another work using 20 mg of Ag/Pd@UiO‐66‐NH_2_ catalyst reports on ∼39.4 μmol h^−1^ [30], compared with our 8.7 μmol h^−1^ obtained with 5 mg of MOF. A comparison with more works using similar MOFs is given in Table S1. When the reaction was performed in the presence of AgNO_3_, H_2_O_2_ formation was almost completely suppressed (only ∼170 µmol g^−1^), demonstrating that H_2_O_2_ formation proceeds predominantly through reduction of dissolved molecular oxygen by photogenerated electrons. Introducing benzoquinone (BQ), which acts as a superoxide (*O_2_^−^) scavenger and possesses a redox potential comparable to that of the O_2_/*O_2_^−^ couple (∼−0.18 V vs. NHE), resulted in a moderate decrease in H_2_O_2_ production (∼950 µmol g^−1^ compared with ∼1350 µmol g^−1^). The relatively modest suppression by BQ could be the explained by the tendency of BQ to operate as an electron scavenger by attacking *O_2_^−^. Here, although superoxide intermediates may participate in the reaction pathway, the rapid delivery of the second electron required for H_2_O_2_ formation from the OOH* intermediate limits the accumulation of *O_2_^−^, thereby reducing the effectiveness of BQ as a scavenger [31].

The photocatalytic stability of the most active catalyst, 7%Cu@UiO‐66(Zr)‐NH_2_, was evaluated through repeated photocatalytic cycles (Figure S9C). After three consecutive reaction cycles, only a slight decrease in H_2_O_2_ yield was observed. This minor reduction is primarily attributed to the partial loss of catalyst during the recovery and washing processes, indicating that the material maintains good structural stability and reusability under the applied reaction conditions. Furthermore, structural analysis through FTIR of the recovered catalyst reveals that the characteristic framework features remain largely preserved after the recycling experiments (Figure S9D). This observation confirms that the 7%Cu@UiO‐66(Zr)‐NH_2_ framework retains its crystallinity and structural integrity during repeated photocatalytic operation. While the current study was performed in laboratory settings, studies carried out under conditions of changing pH, real water media, and differing anions can be useful for determining the application of the photocatalyst [32, 33].

## 3 Discussion

**FIGURE 14:**
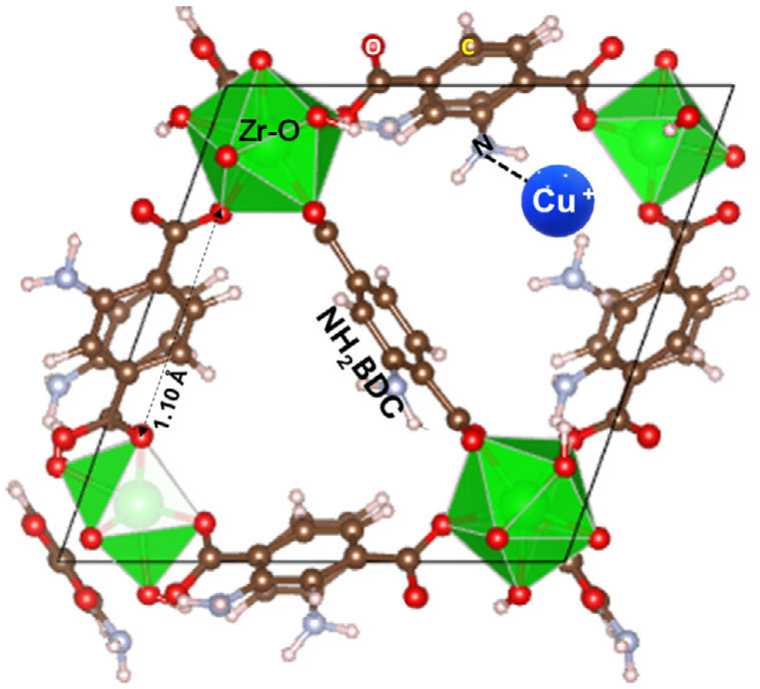
Schematic representation of the Copper coordination with UiO‐66(Zr)‐NH_2_.

A consolidated analysis of the results reveals that copper is predominantly stabilized in the +1‐oxidation state within UiO‐66(Zr)‐NH_2_ and is incorporated inside the framework's pores through interaction with the amino groups of the organic linker, as inferred from our XPS results. Here, it should be noted that Cu^+^ and Cu^2+^ cations may also be coordinated to the OH groups of the IBU as previously reported [34]. The preparation method can therefore significantly influence the location of copper species and, consequently, the catalytic activity. Although copper was introduced as Cu^2+^ in the synthesis procedure, it was preferentially reduced and is stabilized as Cu^+^ due to ligand‐assisted reduction by the electron‐rich NH_2_‐functionalized linker (Figure 14) [5, 35]. The resulting Cu^+^ species are stabilized through coordination with nitrogen donor atoms from the amino groups of the linkers. This creates a Cu—N coordination environment that helps maintain copper in the monovalent state and reduces its tendency to oxidize or disproportionate. At the same time, the porous structure of the MOF provides spatial confinement next to the Zr–O units, which limits the mobility of copper species and prevents their aggregation into metallic clusters or CuO_*x*_ particles [36]. As a result, copper remains dispersed as isolated Cu^+^ sites within the framework, preserving its electronic properties and catalytic functionality. Similar Cu^+^ stabilization via nitrogen coordination and pore confinement has been widely reported in MOF and N‐rich catalytic systems [37, 38]. Furthermore, the absence of new absorption bands, emission features, or spectral shifts indicates that copper remains highly dispersed and does not form Cu‐(hydr)oxide nanoparticles.

Electronically, the introduction of copper reduced the apparent bandgap, as found also by other groups [14]. Another remarkable effect of incorporating copper is an irreversible charge transport to the copper ion, driven by thermodynamics, since the LUCO of UiO‐66(Zr)‐NH_2_ is located approximately at −0.6 V relative to NHE, whereas the redox level of Cu^+^ is located at 0.52 V. A key mechanistic question concerns the pathway by which photoexcited electrons reach the Cu centers in Cu@UiO‐66(Zr)‐NH_2_. Two possibilities can be considered: a sequential ligand → Zr → Cu charge transfer or a direct ligand→Cu charge transfer. The absence of electronic coupling between Zr and Cu, as evidenced by invariant Zr 3*d* binding energies and unchanged Zr‐carboxylate vibrational signatures in XPS and FTIR, rules out efficient Zr‐mediated transfer. Therefore, direct ligand‐to‐Cu charge transfer seems to be the dominant. As a result, two competitive excited‐state pathways coexist in Cu‐modified UiO‐66(Zr)‐NH_2_: the intrinsic ligand‐to‐Zr_6_‐oxo charge transfer and a newly introduced ligand‐to‐Cu^+^ pathway.

**FIGURE 15:**
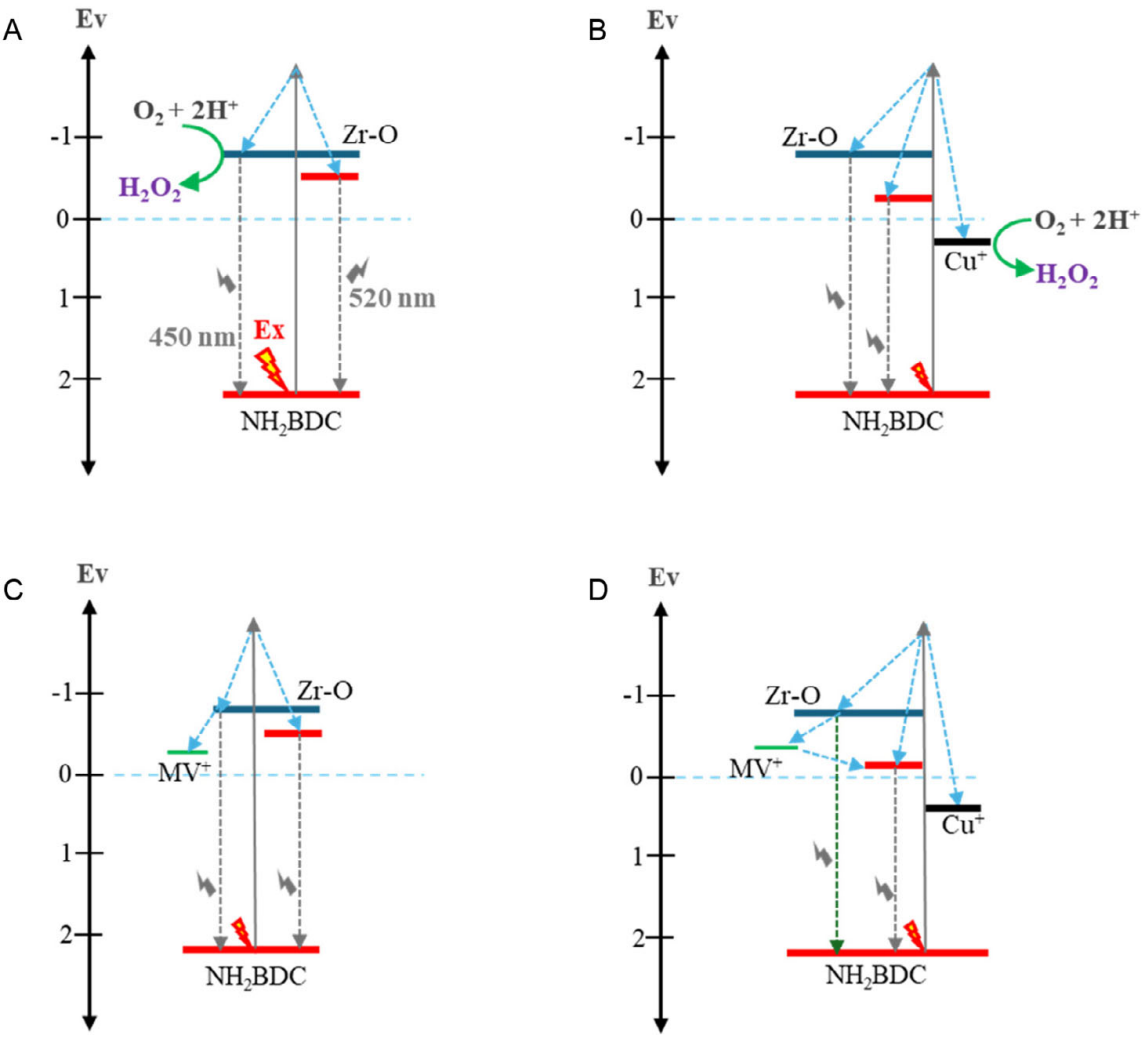
Band diagram illustrating the presence of different emission states observed during the PL experiment in (A) Pristine UiO‐66(Zr)‐NH_2_, (B) Cu@UiO‐66(Zr)‐NH_2_, (C) UiO‐66(Zr)‐NH_2_ in the presence of MV^+^, and (D) Cu@UiO‐66(Zr)‐NH_2_ in the presence of MV^+^. The band positions in the figure are based combining our findings with published data [39, 40, 41].

The PL measurements support this scenario. The PL signal can be resolved into three emissive peaks at 3.05 eV (406 nm), 2.76 eV (449 nm), and 2.4 eV (520 nm) (Figure 15A). The dominant emission at 2.76 eV is attributed to ligand‐to‐ cluster charge–transfer (LCCT) recombination, involving the {Zr_6_O_4_(OH)_4_} node, while the 3.05 and 2.43 eV bands are associated with ligand‐localized excited states, as inferred from the PL measurements of NH_2_‐BDC linker. The relatively small energy separations between these states (∼0.29–0.33 eV) allow vibrational redistribution of excited electrons, which is manifested by complete overlap in the PL envelope of the pristine UIO‐66(Zr)‐NH_2_ MOF. Upon Cu incorporation, a substantial suppression of the radiative recombination of the 2.76 eV (∼450 nm) peak takes place, due to the partial closure of the ligand to Zr—O charge transfer avenue. Consequently, resolving the other two peaks becomes easier. Despite the need for additional photoelectrochemical studies such as transient photocurrent, EIS, and Mott‐Schottky to validate the mechanistic explanation, the overall combination of PL, TRPL, TRIR, and variable temperature FTIR experiments clearly confirms that Cu regulates the charge separation process in the excited state of the UiO‐66(Zr)‐NH_2_ material [42].

A noteworthy observation in the PL measurements was the emergence of an emission band at 520 nm, but only when methyl viologen (MV) was present as an electron scavenger. As noted above, this band coincides with the characteristic emission of the NH_2_‐BDC ligand and can therefore be assigned to intra‐ligand radiative recombination. Interestingly, this emission is absent in pristine UiO‐66(Zr)‐NH_2_, despite the presence of MV, while its intensity increases progressively with increasing Cu incorporation in the MOF. This behavior is particularly intriguing and suggests that the presence of copper plays a decisive role in enabling this emissive pathway.

To rationalize this observation, it is instructive to examine the energy‐level diagram of the system (Figure 15). In this diagram, the highest occupied crystal orbital (HOCO) and lowest unoccupied crystal orbital (LUCO) positions are taken from published reports [39], as are the redox potentials of Cu^+^/Cu and MV^2+^/MV^+^ [40, 41]. The photoluminescence (PL) emitting levels were estimated from the emission energies together with the position of the HOCO level. As shown in the diagram, the Cu^+^ level lies substantially lower in energy than the 520 nm emissive state. Consequently, back‐electron transfer from Cu to the 520 nm emitting state is energetically highly unfavorable. Therefore, population of the 520 nm emissive state must occur via methyl viologen (MV), which acts as an electron mediator. The necessity of MV is further supported by the complete absence of the 520 nm emission when MV is not present. However, this raises an important question: If MV is required to mediate this process, and Cu simultaneously competes with MV for photogenerated electrons, why is the 520 nm emission not observed for the pristine MOF even in the presence of MV?

To the best of our understanding, this apparent inconsistency can be rationalized by considering that Cu incorporation does not merely act as an electron sink but also modifies the electronic structure of the MOF. Specifically, Cu incorporation leads to a reduction in the band gap, shifting the LUCO level downward and consequently altering the position of the NH_2_‐BDC emissive state relative to the MV redox level. The redox potential of the MV^2+^/MV^+^ couple is −0.44 V versus NHE, which corresponds to an energy level slightly lower than that of the 520 nm emissive state. In pristine UiO‐66(Zr)‐NH_2_, electrons transferred from the Zr—O clusters to MV are therefore unlikely to return to the NH_2_‐BDC ligand due to unfavorable energetics. Upon Cu incorporation, however, the downward shift of the LUCO also shifts the NH_2_‐BDC emissive state to lower energy. This shift becomes sufficient to render electron transfer from MV^+^ back to the NH_2_‐BDC ligand energetically feasible, ultimately enabling radiative recombination that gives rise to the 520 nm emission. It should be noted that the 520 nm emission originates from electrons, initially excited within the {Zr_6_O_4_(OH)_4_} clusters. Although these electrons represent only a minor fraction of the excited carriers‐since a significant portion of the photogenerated electrons are captured by Cu centers, partially suppressing the 450 nm PL signal. So, their emission remains observable due to the absence of stronger competing emission pathways.

Three key observations emerge from the TRIR measurements. First, pristine UiO‐66(Zr)‐NH_2_ exhibits a broad transient mid‐IR absorption that partly originates from photoinduced lattice heating, as confirmed by comparison with variable‐temperature FTIR spectra. Second, this transient baseline gradually diminishes upon Cu incorporation. Third, the disappearance of the transient feature associated with the {Zr_6_O_4_(OH)_4_} units in the presence of electron scavengers across all MOF samples confirms that this signal originates from photogenerated electrons. Taken together, these results demonstrate that Cu incorporation suppresses nonradiative thermal dissipation pathways while promoting more efficient charge extraction.

The modification of excited‐state charge dynamics directly influences the photocatalytic oxygen reduction reaction (ORR) responsible for H_2_O_2_ production. Photocatalytic ORR may proceed via a direct two‐electron reduction pathway (O_2_ + 2H^+^ + 2e^−^ → H_2_O_2_) or through an indirect two‐step single‐electron pathway involving superoxide radicals (O_2_ + e^−^ → *O_2_^−^; *O_2_^−^+ e^−^ + 2H^+^ → H_2_O_2_). A competing four‐electron reduction pathway (O_2_ + 4H^+^ + 4e^−^ → 2H_2_O) consumes oxygen without producing H_2_O_2_ and therefore reduces selectivity [43]. The efficiency of photocatalytic H_2_O_2_ generation is therefore governed by the ability of the catalyst to favor the two‐electron reduction pathway while suppressing the four‐electron process.

In Cu‐modified UiO‐66(Zr)‐NH_2_, the introduction of Cu^+^ centers alters both the electronic and adsorption properties of the catalyst surface. Temperature‐dependent FTIR measurements indicate that Cu incorporation decreases surface water adsorption. In parallel, the addition of copper is likely to enhance O_2_ adsorption, due to the strong affinity of Cu^+^ toward molecular oxygen through *π*‐back‐donation interactions, enabling the formation of Cu^+^‐O_2_ adducts that facilitate oxygen activation [44, 45]. The fact that Cu^+^ centers favor associative O_2_ adsorption, where the O—O bond remains intact, is in favor of obtaining H_2_O_2_. PL and TRIR analyses demonstrate that Cu^+^ sites function as efficient electron‐accumulation centers, capturing photogenerated electrons from the excited NH_2_‐BDC linker through ligand‐to‐Cu charge transfer thus preventing thermal dissipation. The localized accumulation of electrons at these Cu centers promotes the reduction of adsorbed O_2_ molecules, forming superoxide radicals (*O_2_^−^) via single‐electron transfer. These superoxide intermediates subsequently form OOH then undergo a second electron‐transfer step to eventually yield H_2_O_2_ through the two‐step single‐electron ORR pathway.

## 4 Conclusion

Cu incorporation into UiO‐66(Zr)‐NH_2_ generates site‐isolated Cu^+^ centers that interact with the amino‐functionalized linker while preserving the structural integrity of the {Zr_6_O_4_(OH)_4_} framework. Spectroscopic results show that these Cu sites capture photogenerated electrons, altering the excited‐state relaxation dynamics and suppressing vibrational energy dissipation observed in pristine UiO‐66(Zr)‐NH_2_. The localized electron density at Cu centers enhances oxygen activation and promotes the formation of reactive intermediates responsible for H_2_O_2_ generation. These results demonstrate that introducing isolated metal centers can effectively regulate charge–transfer processes and improve photocatalytic oxygen reduction within MOF frameworks.

## 5 Experimental Section

### 5.1 Chemicals

2‐Aminoterephthalic acid (99%, Thermo Scientific) (BDC‐NH_2_), zirconium (IV) propoxide solution (70 wt% in 1‐propanol), and copper (II) nitrate trihydrate (≥99%) were purchased from Sigma–Aldrich. Glacial acetic acid (≥99.7%), *N*,*N*‐dimethylformamide (DMF, ≥99.8%), and absolute ethanol (≥99.8%) were purchased from Carlo Erba. All chemicals were used as received without further purification for the synthesis procedures. For ICP analysis, zirconium and copper standard solutions (1000 mg L^−1^, TraceCERT, Sigma–Aldrich), hydrochloric acid (HCl, 37 wt%, ISO grade, Sigma–Aldrich), and sodium bicarbonate (≥99.7%, Sigma–Aldrich) were used.

### 5.2 Synthesis of UiO‐66(Zr)‐NH_2_

Following a reported procedure [46, 47], 3.550 mL of a 70% zirconium propoxide solution in 1‐propanol (7.97 mmol), 350 mL of DMF, and 200 mL of acetic acid were added to a 1 L screw‐cap glass bottle. The solution was heated in an oil bath at 130°C for 2 h, during which a noticeable color change from colorless to transparent yellow was observed, indicating the formation of the {Zr_6_O_4_(OH)_4_} cluster. The mixture was then allowed to cool to room temperature. After cooling, a magnetic stirring bar was added along with 4.117 g (22.7 mmol) of BDC‐NH_2_, and the solution was stirred at room temperature for 18 h at 300 rpm. The resulting solid, beige in color, was recovered by centrifugation at 13 000 rpm for 10 min, washed with approximately 500 mL of DMF to remove excess ligand, and subsequently with around 500 mL of hot absolute ethanol to eliminate residual DMF. The obtained UiO‐66(Zr)‐NH_2_ could be stored in absolute ethanol as a colloidal suspension for further use or dried at 100°C in air to a powdered form. This procedure produces approximately 1.8 g of pure MOF (80% yield).

### 5.3 Synthesis of 2, 4, and 7 wt% Cu@UiO‐66(Zr)‐NH_2_

Cu@UiO‐66(Zr)‐NH_2_ composites were prepared by a wet‐impregnation method [48]. A concentrated Cu^2+^ solution (0.4 g mL^−1^) in absolute ethanol was prepared using Cu(NO_3_)_2_·3H_2_O as the Cu^2+^ precursor, and a colloidal suspension of UiO‐66(Zr)‐NH_2_ in absolute ethanol (25 mg mL^−1^) was also prepared. To obtain a final amount of approximately 200 mg of each composite, 7.88 mL of the MOF suspension was mixed with 0.029 mL of the Cu^2+^ solution for the 2 wt% sample, 7.68 mL of the MOF suspension was mixed with 0.080 mL of the Cu^2+^ solution for the 4 wt% sample, and 7.44 mL of the MOF suspension was mixed with 0.133 mL of the Cu^2+^ solution for the 7 wt% sample. The mixtures were stirred at room temperature for 30 min and then centrifuged at 13 000 rpm for 10 min. The resulting solids were washed once with 20 mL of absolute ethanol and dried overnight at 100°C, followed by a post‐thermal treatment under vacuum at 150°C for 12 h. The final Cu content in the composites was estimated by SEM‐EDX and more precisely quantified by ICP‐OES analysis.

### 5.4 Characterization Methods

Routine powder X‐ray diffraction (PXRD) patterns were recorded at room temperature with a D5000S X’Pert MDP diffractometer (*λ*Cu, Kα1, Kα2) from 5° to 50° (2*θ*) using a step of 0.04° and 20 s of accumulation per step in continuous mode. Scanning electron microscopy (SEM) images were obtained using a JEOL JSM‐7001F microscope. Samples were sputter‐coated with gold prior to analysis. Energy‐dispersive X‐ray (EDX) spectra were acquired using an X‐Max silicon drift detector at an accelerating voltage of 15 kV, with a collection time of 120 s and total counts exceeding 10 000. Nitrogen adsorption–desorption isotherms were measured at 77 K using a Micromeritics TriStar instrument. Samples were pre‐activated under vacuum at 150°C for 5 h prior to analysis.

Diffuse reflectance UV–vis spectra were recorded on a Shimadzu UV‐2600 spectrophotometer equipped with an ISR‐2600 Plus integrating sphere, using BaSO_4_ as the reflectance standard. Fluorescence lifetimes were measured using the time‐correlated single‐photon counting (TCSPC) technique on a CHIMERA spectrometer (Light Conversion) with excitation at 355 nm. A Yb‐based PHAROS laser (Light Conversion; pulse duration 190 fs) coupled to an ORPHEUS optical parametric amplifier was employed as the excitation source. Emission decay was monitored at 450 nm using a hybrid detector (Becker & Hickl HPM‐100‐07), with an instrument response function (IRF) below 50 ps (FWHM). High‐resolution transmission electron microscopy (HRTEM) was performed on a Thermo Scientific Talos F200X G2 microscope operated at 200 kV and equipped with an energy‐dispersive X‐ray detector for nanostructural and compositional analysis. X‐ray photoelectron spectroscopy (XPS) measurements were conducted using a PHI 5000 VersaProbe III spectrometer with a 5 keV focused X‐ray beam to determine surface elemental composition and oxidation states. A metal foil was used for calibration. High‐angle annular dark‐field scanning transmission electron microscopy (STEM‐HAADF) imaging and X‐ray energy‐dispersive spectroscopy (XEDS) mapping were performed on a JEOL JEM‐2100F microscope (IMPMC, Paris, France) operating at 200 kV, equipped with a field‐emission gun and an ultrathin‐window detector enabling light‐element detection, allowing Z‐contrast imaging in HAADF mode.

Inductively coupled plasma optical emission spectroscopy (ICP‐OES) was performed using an Agilent 720 Series instrument to determine the copper content in the composites. The samples were digested in a 1 M NaHCO_3_ solution, using a 5 mg/mL ratio, and heated at 80°C for 16 h. Under these conditions, complete mineralization was achieved for the pristine UiO‐66(Zr)‐NH_2_. However, in the case of Cu‐containing composites, a precipitate was observed during digestion, which was attributed to the formation of Cu(OH)_2_ or related copper hydroxide species. To fully dissolve it, HCl was added following the initial digestion step. Copper emission intensity was measured at 324.7 nm using external calibration with standard Cu solutions. All samples were analyzed in triplicate to ensure reproducibility.

Steady‐state FTIR spectra were recorded at room temperature using a Bruker V70 spectrophotometer. Samples were prepared by dispersing 0.5 wt% photocatalyst in spectroscopy‐grade KBr (Fisher Scientific) and grinding the mixture to a fine powder. The resulting powder was pressed into thin pellets under a load of 5 tonnes for 1 h. Both room‐temperature and elevated‐temperature steady‐state FTIR measurements were conducted in air using a deuterated triglycine sulfate (DTGS) detector. For temperature‐dependent measurements, the pellet was mounted in a temperature‐controlled sample holder positioned within the spectrometer compartment.

Transient infrared (TRIR) measurements were performed on the same Bruker V70 spectrophotometer coupled to an Nd:YAG pulsed laser (Q‐smart 450, Quantel, UK; 5 ns pulse width, 10 Hz repetition rate). The experiments were carried out using a step‐scan acquisition mode [49]. Excitation was carried out using the third harmonic (355 nm) of the Nd:YAG laser. Transient IR signals were detected using a fast, spectrally extended mercury cadmium telluride (MCT) detector, enabling measurements down to 550 cm^−1^ with a temporal resolution of 2.5 ns. The signal was recorded and averaged over 20 laser pulses at each interferometer mirror position. An excitation energy of 10 mJ per pulse was used, controlled by appropriate attenuating filters (Kopp 5860 and 5840). The entire IR optical path was maintained under vacuum to enhance the signal‐to‐noise ratio. Samples were prepared by pressing a homogeneous mixture of 2.1 mg KBr containing 0.25 wt% MOF under 5 tonnes for 60 min to form a uniform pellet. Additional details of the experimental setup are described elsewhere [50, 51]. Methyl viologen (MV) was employed as an electron scavenger to probe its effect on the transient IR signals. For these measurements, 2 mg of MV was thoroughly ground together with 0.5 mg of the powdered sample and 210 mg of KBr. The homogeneous mixture was then pressed into a pellet following the procedure described above.

### 5.5 Photocatalytic H_2_O_2_ Production Test

In a typical batch photocatalytic experiment, 5 mg of the powdered catalyst was uniformly dispersed in 20 mL of a 10 vol% methanol–deionized water mixture (18 ml water + 2 ml MeOH) in a photochemical reactor. Prior to irradiation, the suspension was continuously purged with high‐purity gas (air/N_2_) for 30 min. The reaction mixture was then irradiated for 90 min using a simulated solar light source (Model 10 500 solar simulator, ABET Technologies, USA) with an illumination area of 19.6 cm^2^ (photon flow app. 7.4 × 10^17^ photons/sec, assuming 2.75 eV for an average photon). After irradiation, the catalyst particles were separated from the reaction mixture by centrifugation, and the resulting clear solution was collected for analysis. The concentration of generated H_2_O_2_ was determined using a colorimetric iodometric method. Briefly, 1 mL of the reaction solution was mixed with 2 mL of acidic KI indicator solution and allowed to react for 30 min to ensure complete oxidation of iodide. The absorbance of the resulting solution was then measured using UV–Vis spectroscopy at ∼350 nm [52], and the H_2_O_2_ concentration was calculated based on a previously established calibration curve (Figure S9A,B). For the scavenger experiments, 1 mM of AgNO_3_ and, alternatively, 1 mM of benzoquinone (BQ) were used as electron scavengers. These experiments were conducted using the most active photocatalyst under identical reaction conditions. Also, blank AgNO_3_ and BQ solutions were used as background references during the analysis to eliminate possible ambiguity arising from their intrinsic absorption or coloration effects. For the recyclability tests, the catalyst was recovered after each reaction cycle by centrifugation, thoroughly washed with deionized water to remove residual reactants and intermediates, and subsequently dried at 100°C before being reused in the next photocatalytic run.

## Funding

This study was supported by the H2020 Energy (101022649 and 101084131).

## Conflicts of Interest

The authors declare no conflicts of interest.

## Supporting information

Supplementary Material

## Data Availability

The data that support the findings of this study are available on request from the corresponding author. The data are not publicly available due to privacy or ethical restrictions.
