# Supplementary material for: Regulating Ligand‐to‐Metal Charge Transfer in UiO‐66(Zr)‐NH2 With Site‐Isolated Cu+ Centers for Efficient Photocatalytic H2O2 Generation
Source: Small Sci. 2026 Jul 30;6(8):e70333. doi: 10.1002/smsc.70333 (PMC13431963; doi:10.1002/smsc.70333)
Supplement: Supplementary file 1 — Supplementary Material [file SMSC-6-e70333-s001.pdf]

Regulating Ligand-to-Metal Charge Transfer in UiO-66-NH_2 with Site-Isolated Cu^+-Centers for Efficient Photocatalytic H_2O_2 Generation

Aneek Kuila[1], Khaled Dassouki[2], Aditya Swarup Lal[3], Ambili Ramanthrikkovil Variyam[4], Nirmalendu Sekhar Mishra[5], Eddy Dumas[2], Nadav Amdursky[4, 6], Nathalie Steunou[2], Yaron Paz [1*]

[1] Department of Chemical Engineering, Technion, 3200003 Haifa, Israel.

*Corresponding author. E-mail : paz@technion.ac.il

[2] Université Paris-Saclay, UVSQ, UMR CNRS 8180, Institut Lavoisier de Versailles, 78000 Versailles, France

[3] Environmental Nanotechnology Laboratory, Department of Environmental Science and Engineering, Indian Institute of Technology (ISM), Dhanbad, 826004, Jharkhand, India

[4] Schulich Faculty of Chemistry, Technion-Israel Institute of Technology, Haifa 3200003, Israel

[5] Green Energy & Applied Research (G.E.A.R) Lab, Department of Environmental Science and Engineering, SRM University-AP, Amaravati, Andhra Pradesh, 522240, India

[6] Chemistry, School of Mathematical and Physical Sciences, University of Sheffield, Sheffield S3 7HF, United Kingdom

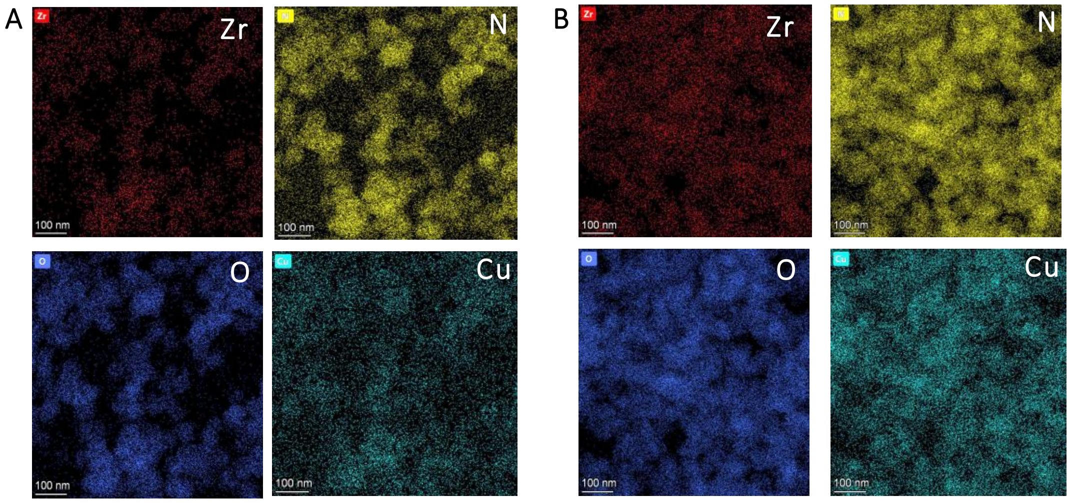

Figure S1: TEM-EDS mapping of the B) 2%Cu@ UIO-66(Zr)-NH_2 and B) 7%Cu@ UIO-66(Zr)-NH_2

a

b

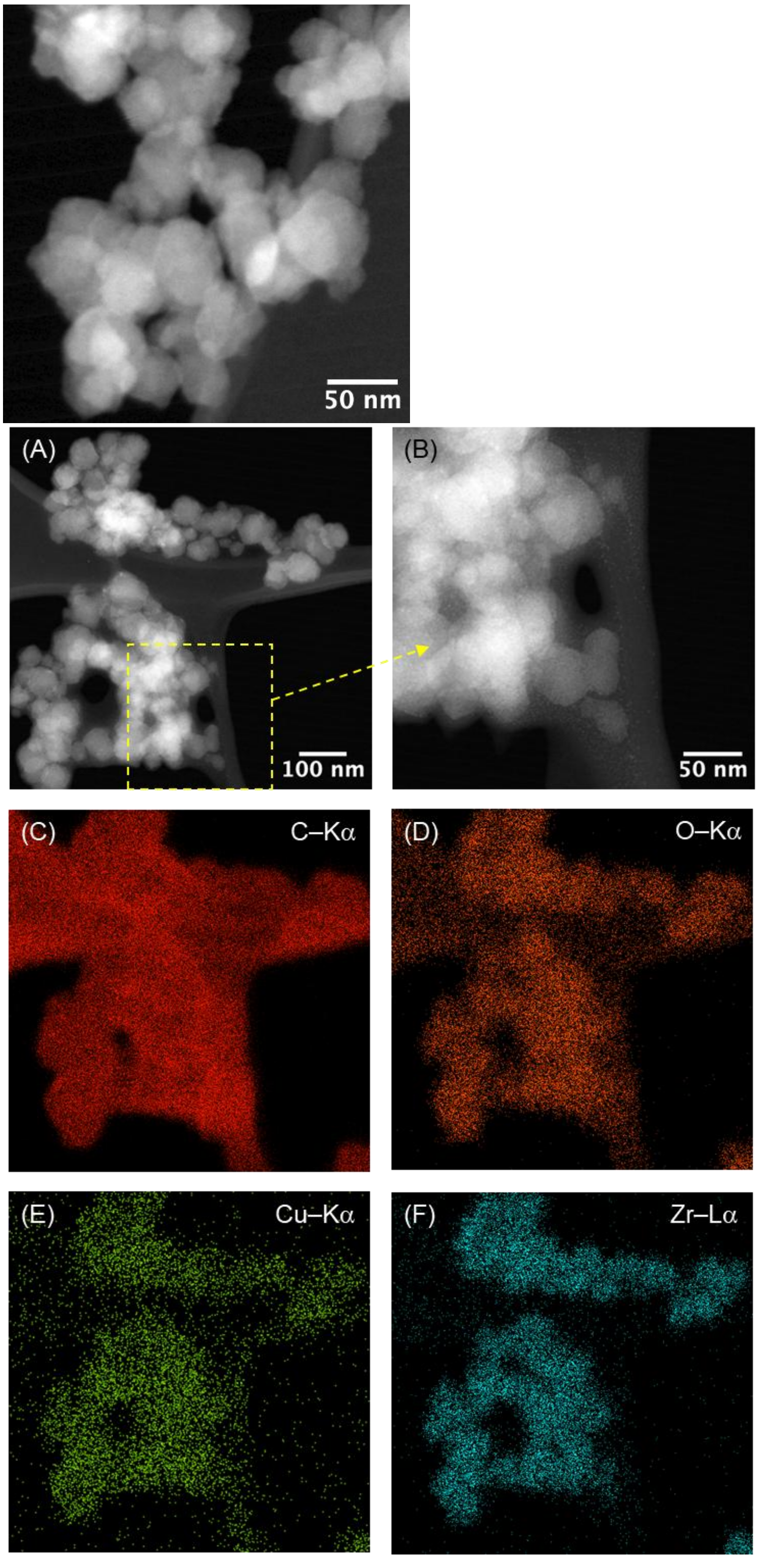

Figure S2 a) HAADF-STEM image of UIO-66(Zr)-NH_2; b) characterization of 7%Cu@ UIO-66(Zr)-NH_2 A,B) HAADF-STEM images and C- F) STEM-XEDS elemental maps corresponding to image A)

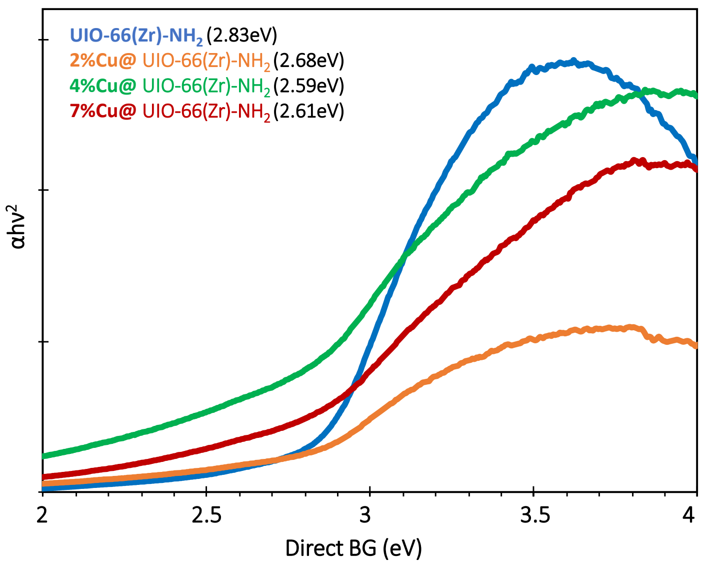

Figure S3. Bandgap calculation of the MOFs

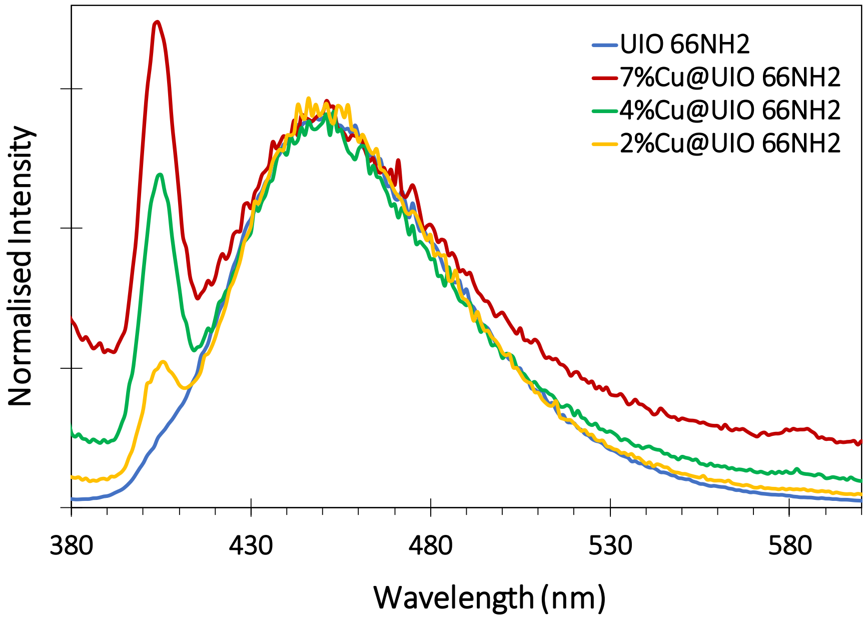

Figure S4. The (normalized) line shape of the PL spectra of UIO-66(Zr)-NH_2

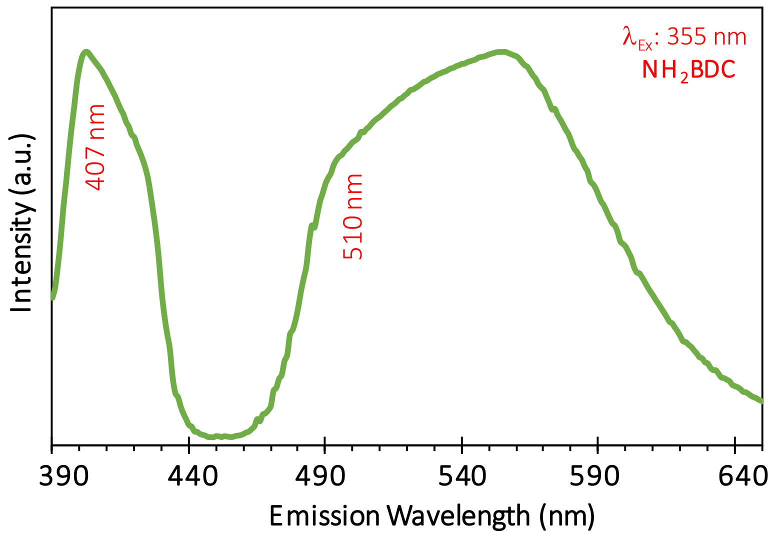

Figure S5. PL emission spectra of pristine NH2BDC ligand after 355 nm excitation

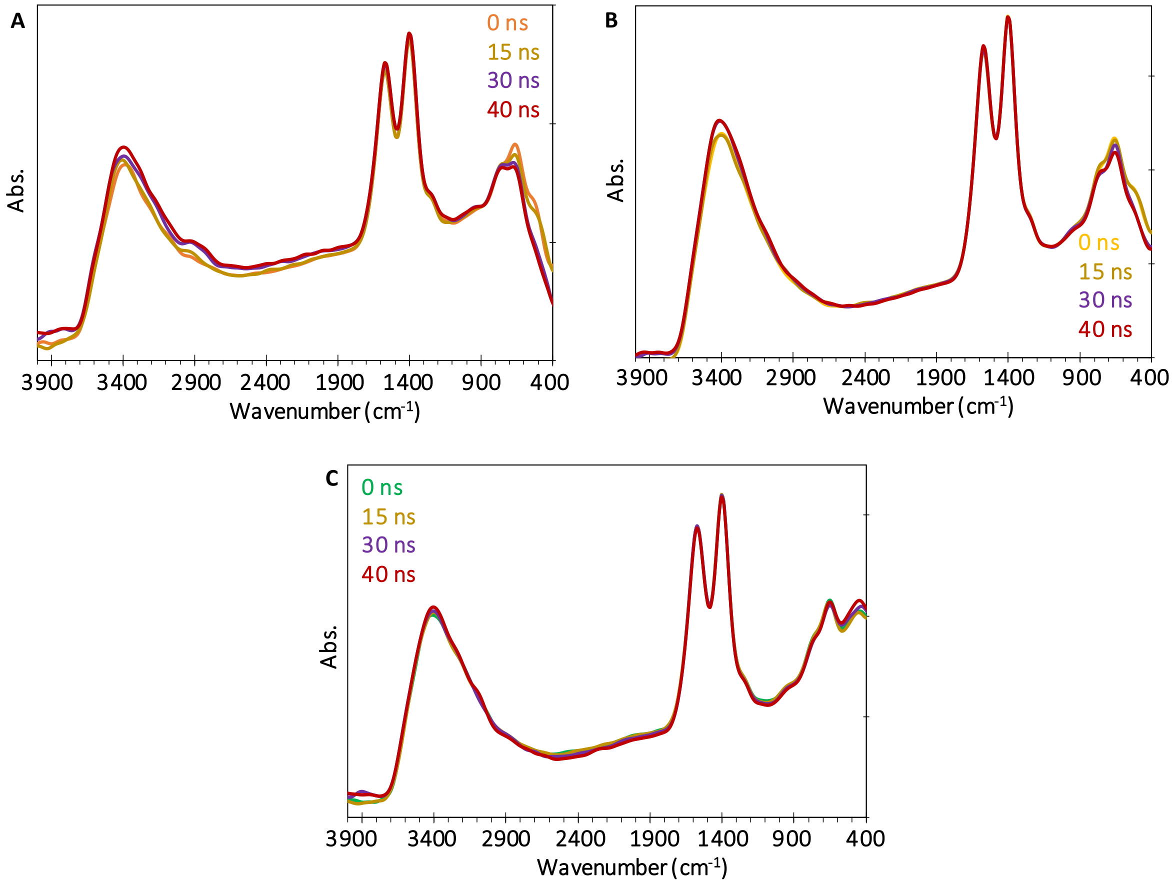

Figure S6. TRIR spectra of A) 2%Cu@ UIO-66(Zr)-NH_2 B) 4%Cu@ UIO-66(Zr)-NH_2, C) 7%Cu@ UIO-66(Zr)-NH_2 under 355nm excitation

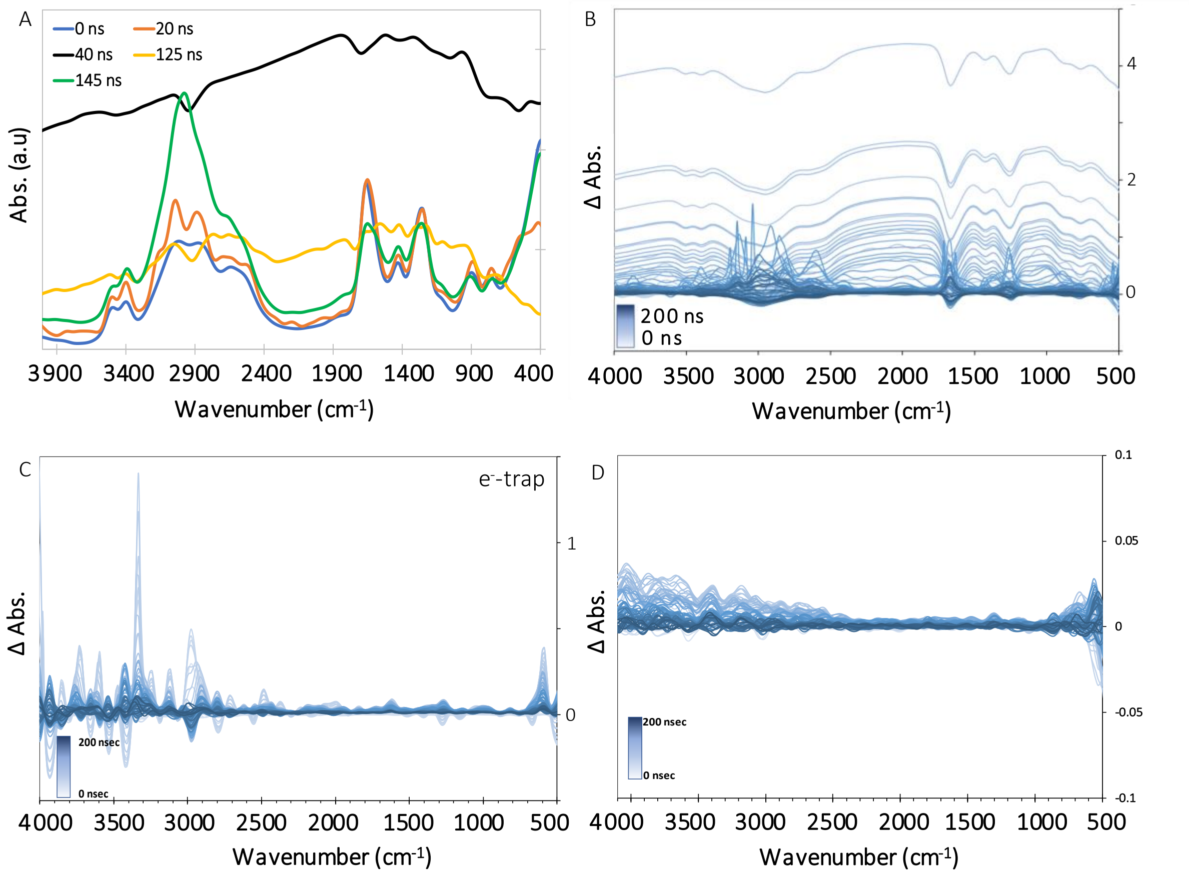

Figure S7. TRIR A) absorbance spectra and B) Δ Abs spectra of NH_2BDC without scavenger. C) TRIR spectra of NH2BDC in the presence of an electron scavenger. D) TRIR Δ Abs spectra of BDC without scavenger

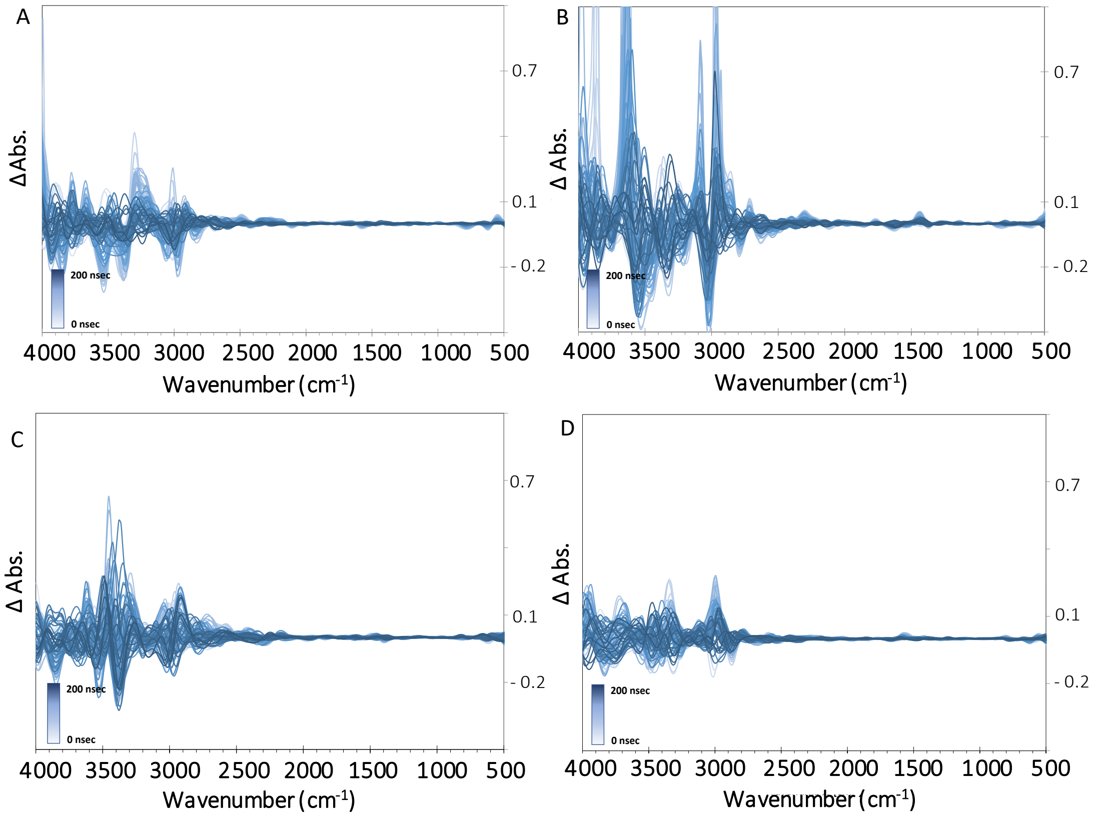

Figure S8. TRIR spectra of the A) UIO-66(Zr)-NH_2, B) 2%Cu@ UIO-66(Zr)-NH_2, C) 4%Cu@ UIO-66(Zr)-NH_2 and D) 7%Cu@ UIO-66(Zr)-NH_2 under the influence of the electron scavenger

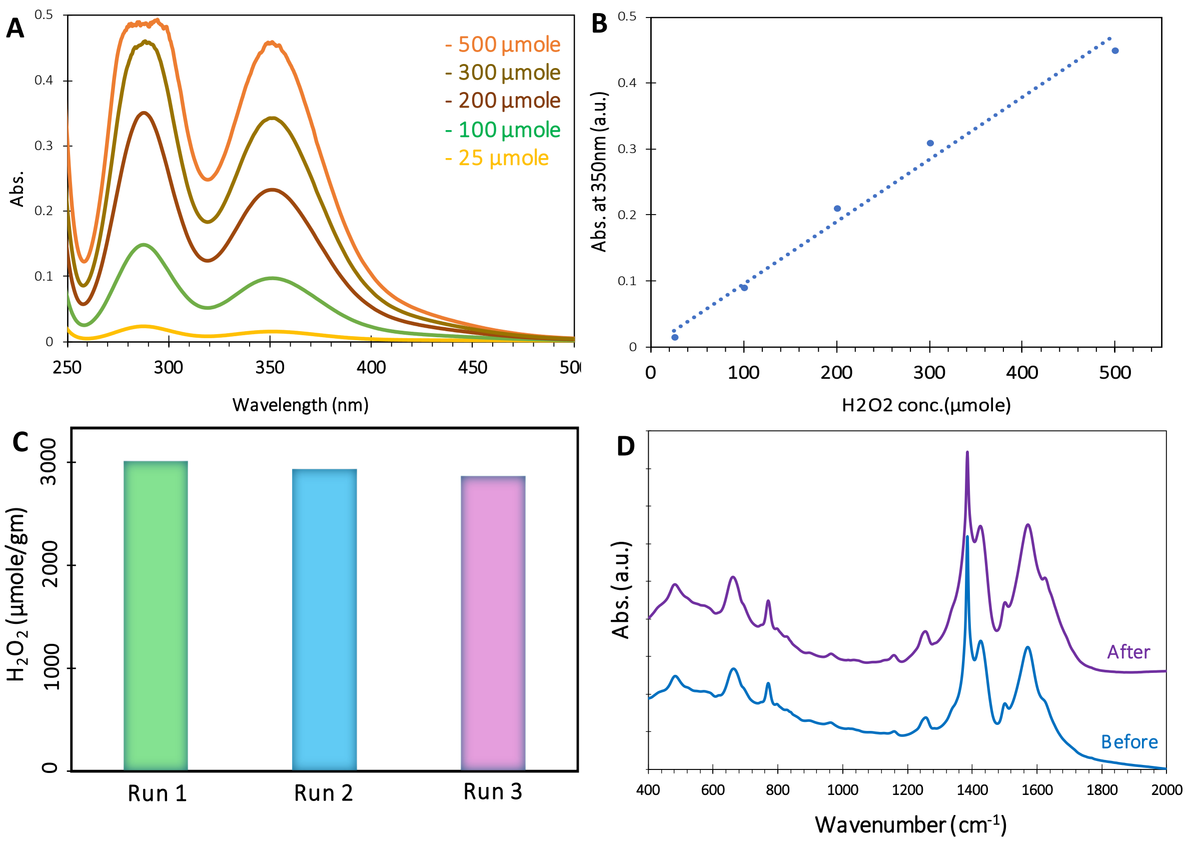

Figure S9: A-B) Calibration curves were constructed using solutions containing known concentrations of H_2O_2 treated with an acidic KI indicator. For all concentrations, the absorbance at 350 nm was used for quantitative analysis. B) Recyclability performance of 7%Cu@UiO-66-NH_2 during photocatalytic H_2O_2 production over successive reaction cycles. and C) Structural stability of 7%Cu@UiO-66-NH_2 after three consecutive reuse cycles, demonstrating preservation of the framework structure.

Table S1: Comparison of previously reported MOF-based photocatalysts for photocatalytic H_2O_2 production under visible-light irradiation, including H_2O_2 production performance, and apparent quantum efficiency (AQE). In the Table, “*” means “no details on irradiance area” and “&” means “no details on photon flux”.

**Photocatalyst**	**Light source**	**Illumination conditions**	**AQE**	**H_2O_2 production performance**	**Ref.**
Ag/Pd@UiO-66-NH_2	Visible light	$\lambda > 420$ nm., 20 mg of catalyst *,&		~39.4 µmol. h^{-1}	1
$ZnIn_2S_4$/UiO-66-NH_2	Visible light	350 mW cm^{-2}, $\lambda > 420$ nm., 20 mg of catalyst *	1.79%	~550 µmol. L^{-1} h^{-1}	2
ZIF-67 derivate (Co_3O_4@CN)	Visible light	17.3 mW cm^{-2}, $\lambda > 420$ nm.,10 mg of catalyst *		2655.3 µmol· g^{-1}. h^{-1}	3
CN/Zn-MOF	Visible light	$\lambda > 400$ nm., 10 mg of catalyst *,&		47.8 µmol. h^{-1}	4
CDs@PCN-222	Vis–NIR light	50 mW cm^{-2}, $\lambda >$ 420 nm.,10 mg of catalyst *		13400 µmol. g^{-1}. h^{-1}	5
Au@MIL-125-NH_2	Visible light	100 mW cm^{-2}, $\lambda > 520$ nm., 5 mg of catalyst *		2160 µmol. g^{-1}. .h^{-1}	6
ZnO/UiO-66-NH_2	Visible light	125 mW cm^{-2}, $\lambda > 420$ nm., 20 mg of catalyst *		789.89 µmol. L^{-1}.h^{-1}	7
Semiconductive conjugated MOF	Visible light	5 mg catalyst,	8.2%	1676 µmol. g^{-1}. h^{-1} (w/o scavenger) 4000 µmol. g^{-1}. h^{-1} (w hole scavenger)	8
UiO-66-B	Visible light	$\lambda > 420$ nm.,30 mg of Catalyst *,&		~1002 µmol. g^{-1}. h^{-1}	9
7%Cu@UiO-66(Zr)-NH_2, (This work)	Visible light	16.6 mW cm^{-2}, $\lambda > 420$ nm.,5 mg catalyst; Photon flow: app. $6.9*10^{17}$ photons s^{-1}		~1630 µmol .g^{-1}. h^{-1} (w hole scavenger)	

References:

[1] S. Dash, S., S.P Tripathy, S. Subudhi, L. Acharya, A. Ray, P. Behera, K. Parida, “Ag/Pd bimetallic nanoparticle-loaded Zr-MOF: an efficacious visible-light-responsive photocatalyst for H_2 O_2 and H_2 production” *Energy Advances*, 3 (2024): 1073-1086, doi: 10.1039/D3YA00597F

[2] J. Qiu, L. Zhang, G. Xia, D. Dai, Y. Tang, J. Yao, “Ligand functionalization on Zr-MOFs enables efficient visible-light-driven H_2O_2 evolution in pure water”, *Catalysis Science & Technology*, 13 (2023): 2101-2107, doi: 10.1039/d3cy00130j.

[3] H. Chen, L. Nie, Y. Yang, C. Fang, X. Chen, X. Li, “ZIF-67-derived Co_3O_4@ CN-assisted g-C_3N_4 for efficient photocatalytic hydrogen peroxide production”, *Molecular Catalysis*, 569 (2024): 114584, 10.1016/j.mcat.2024.114584.

[4] Y. Li, Y. Guo, D. Luan, X. Gu, X.W. Lou, “An unlocked two-dimensional conductive Zn-MOF on polymeric carbon nitride for photocatalytic H_2O_2 production”, *Angewandte Chemie International Edition*, 62 (2023): e202310847, https://doi.org/10.1002/anie.202310847.

[5] Z. Xia, W. Hu, Y. Xiang, C. Lü, “Boron-doped carbon dots modified zirconium porphyrin MOFs enable efficient and highly selective photosynthesis of hydrogen peroxide in water and seawater”, *Applied Catalysis B: Environment and Energy*, 358 (2024): 124394, https://doi.org/10.1016/j.apcatb.2024.124394.

[6] Y. Zhao, H. Ge, Y. Kondo, Z. Guo, Y. Kuwahara, K. Mori, T. Sekino, Z. Bian, H. Yamashita, “Highly reactive facet modulation of Ti-based MOFs by selective anchoring of Au metal for photocatalytic H_2O_2 production”, *ACS Catalysis*, 15 (2025): 11313-11325, ACS Catal. 2025, 15, 11313−11325.

[7] M.A. Qaiser,J. Li, W. Ren, S. Khan, S.B. Ahmed,W.A. Qureshi, M.H. Abdurahman, W. Wang, Q. Liu, “Sacrificial agent-free H_2O_2 production driven by S-scheme photocatalysis over hierarchical ZnO/UiO-66-NH_2 heterojunction. *Chemical Engineering Journal*, 525 (2025):170188, https://doi.org/10.1016/j.cej.2025.170188.

[8] J.Y. Choi, B. Check, X. Fang, S. Blum, H.T. Pham, K. Tayman, J. Park, “Photocatalytic hydrogen peroxide production through functionalized semiconductive metal–organic frameworks”, *Journal of the American Chemical Society*, 146 (2024): 11319-11327, https://doi.org/10.1021/jacs.4c00681.

[9] C.H. Bao, L. Li, X.F. Wang, S.S. Xia, X. Wang, C.C. Jin, & Z. Chen, "Bringing Porous Framework Materials toward Photocatalytic H_2O_2 Production" Nano Letters, 25 (2025): 4115-4136, https://doi.org/10.1021/acs.nanolett.4c06680.
